# The SEK-1 p38 MAP Kinase Pathway Modulates Gq Signaling in *Caenorhabditis elegans*

**DOI:** 10.1534/g3.117.043273

**Published:** 2017-07-07

**Authors:** Jill M. Hoyt, Samuel K. Wilson, Madhuri Kasa, Jeremy S. Rise, Irini Topalidou, Michael Ailion

**Affiliations:** Department of Biochemistry, University of Washington, Seattle, Washington 98195

**Keywords:** p38 MAPK pathway, Gq signaling, animal behavior, *C. elegans*

## Abstract

Gq is a heterotrimeric G protein that is widely expressed in neurons and regulates neuronal activity. To identify pathways regulating neuronal Gq signaling, we performed a forward genetic screen in *Caenorhabditis elegans* for suppressors of activated Gq. One of the suppressors is an allele of *sek-1*, which encodes a mitogen-activated protein kinase kinase (MAPKK) in the p38 MAPK pathway. Here, we show that *sek-1* mutants have a slow locomotion rate and that *sek-1* acts in acetylcholine neurons to modulate both locomotion rate and Gq signaling. Furthermore, we find that *sek-1* acts in mature neurons to modulate locomotion. Using genetic and behavioral approaches, we demonstrate that other components of the p38 MAPK pathway also play a positive role in modulating locomotion and Gq signaling. Finally, we find that mutants in the SEK-1 p38 MAPK pathway partially suppress an activated mutant of the sodium leak channel, NCA-1/NALCN, a downstream target of Gq signaling. Our results suggest that the SEK-1 p38 pathway may modulate the output of Gq signaling through NCA-1(unc-77).

Gq is a widely expressed heterotrimeric G protein that regulates a variety of biological processes, ranging from neurotransmission to cardiovascular pathophysiology (Sánchez-Fernández *et al.* 2014). In the canonical Gq pathway, Gq activates phospholipase Cβ (PLCβ), which cleaves phosphatidylinositol 4,5-bisphosphate into the second messengers diacylglycerol (DAG) and inositol trisphosphate (Rhee 2001). In addition to PLCβ, other Gq effectors have been identified including kinases, such as protein kinase Cζ and Bruton’s tyrosine kinase (Btk) (Bence *et al.* 1997; García-Hoz *et al.* 2010; Vaqué *et al.* 2013), and guanine nucleotide exchange factors (GEFs) for the small GTPase Rho, such as Trio (Williams *et al.* 2007; Vaqué *et al.* 2013). These noncanonical effectors bridge the activation of Gq to other cellular signaling cascades.

In order to study noncanonical pathways downstream of Gq, we used the nematode *Caenorhabditis* *elegans*, which has a single Gαq homolog (EGL-30) and conservation of the other components of the Gq signaling pathway (Koelle 2016). In neurons, EGL-30 signals through EGL-8 (PLCβ) (Lackner *et al.* 1999) and UNC-73 (ortholog of Trio RhoGEF) (Williams *et al.* 2007). UNC-73 activates RHO-1 (ortholog of RhoA), which has been shown to enhance neurotransmitter release through both diacylglycerol kinase (DGK-1)–dependent and –independent pathways (McMullan *et al.* 2006).

To identify additional signaling pathways that modulate Gq signaling, we screened for suppressors of the activated Gq mutant, *egl-30*(*tg26*) (Doi and Iwasaki 2002). *egl-30*(*tg26*) mutant animals exhibit hyperactive locomotion and a “loopy” posture, in which worms have exaggerated, deep body bends and loop onto themselves (Bastiani *et al.* 2003; Topalidou *et al.* 2017). Here, we identify one of the suppressors as a deletion allele in the gene *sek-1*. SEK-1 is a mitogen-activated protein kinase kinase (MAPKK), the *C. elegans* ortholog of mammalian MKK3/6 in the p38 MAPK pathway (Tanaka-Hino *et al.* 2002). The p38 MAPK pathway has been best characterized as a pathway activated by a variety of cellular stresses and inflammatory cytokines (Kyriakis and Avruch 2012). However, the p38 MAPK pathway has also been shown to be activated downstream of a G protein–coupled receptor in rat neurons (Huang *et al.* 2004). Btk, a member of the Tec family of tyrosine kinases, has been shown to act downstream of Gq to activate the p38 MAPK pathway (Bence *et al.* 1997), but *C. elegans* lacks Btk and other Tec family members (Plowman *et al.* 1999).

SEK-1 is activated by the MAPKKK NSY-1 (ortholog of ASK1) and activates the p38 MAPKs PMK-1 and PMK-2 (Andrusiak and Jin 2016). The p38 MAPK pathway, consisting of NSY-1, SEK-1, and PMK-1, is required for innate immunity in *C. elegans* (Kim *et al.* 2002). NSY-1 and SEK-1 are also required for the specification of the asymmetric AWC olfactory neurons (Sagasti *et al.* 2001; Tanaka-Hino *et al.* 2002); the p38 orthologs PMK-1 and PMK-2 function redundantly in AWC specification (Pagano *et al.* 2015). For both innate immunity and AWC specification, the p38 MAPK pathway acts downstream of the adaptor protein TIR-1 (an ortholog of SARM) (Couillault *et al.* 2004; Chuang and Bargmann 2005). Here, we show that the pathway consisting of TIR-1, NSY-1, SEK-1, PMK-1, and PMK-2 also acts to modulate locomotion downstream of Gq signaling.

## Materials and Methods

### C. elegans strains and maintenance

All strains were cultured using standard methods and maintained at 20° (Brenner 1974). The *sek-1*(*yak42*) mutant was isolated from an ENU mutagenesis suppressor screen of the activated Gq mutant, *egl-30*(*tg26*) (Ailion *et al.* 2014). *sek-1*(*yak42*) was outcrossed away from *egl-30*(*tg26*) before further analysis. Double mutant strains were constructed using standard methods (Fay 2006), often with linked fluorescence markers (Frokjaer-Jensen *et al.* 2014) to balance mutations with subtle visible phenotypes. Supplemental Material, Table S1 in File S1 contains all of the strains used in this study.

### Mapping

*yak42* was mapped using its slow locomotion phenotype and its *egl-30*(*tg26*) suppression phenotype. *yak42* was initially mapped to the X chromosome using strains EG1000 and EG1020, which carry visible marker mutations. These experiments showed that *yak42* was linked to *lon-2*, but it was at least several map units (cM) away. *yak42* was further mapped to ∼1 cM away from the red fluorescence insertion marker *oxTi668*, which is located at +0.19 cM on the X chromosome.

### Whole-genome sequencing

Strain XZ1233 *egl-30*(*tg26*); *yak42* was used for whole-genome sequencing to identify candidate *yak42* mutations. XZ1233 was constructed by crossing a *yak42* strain outcrossed two times, back to *egl-30*(*tg26*). Thus, in XZ1233, *yak42* has been outcrossed three times from its original isolate. DNA was isolated from XZ1233 and purified according to the Hobert Laboratory protocol (http://hobertlab.org/whole-genome-sequencing/). Ion torrent sequencing was performed at the University of Utah DNA Sequencing Core Facility. The resulting data contained 10,063,209 reads of a mean read length of 144 bases, resulting in ∼14× average coverage of the *C. elegans* genome. The sequencing data were uploaded to the Galaxy web platform, and we used the public server at usegalaxy.org to analyze the data (Afgan *et al.* 2016). We identified and annotated variants with the Unified Genotyper and SnpEff tools, respectively (DePristo *et al.* 2011; Cingolani *et al.* 2012). We filtered out variants found in other strains we sequenced, leaving us with 605 homozygous mutations. The X chromosome contained 94 mutations: 55 SNPs and 39 indels. Of these, four SNPs were nonsynonymous mutations in protein-coding genes, but only two were within 5 cM of *oxTi668*. However, we were unable to identify *yak42* from the candidate polymorphisms located near *oxTi668*. Transgenic expression of the most promising candidate *pcyt-1* did not rescue *yak42*. Instead, to identify possible deletions, we scrolled through 2 MB of aligned reads on the UCSC Genome Browser, starting at −4.38 cM and moving toward the middle of the chromosome (0 cM), looking for regions that lacked sequence coverage. We found a 3713-bp deletion that was subsequently confirmed to be the *yak42* causal mutation, affecting the gene *sek-1* located at −1.14 cM.

### Locomotion assays

Locomotion assay plates were made by seeding 10 cm nematode growth medium plates with 150 μl of an *Escherichia coli* OP50 stock culture, spread with sterile glass beads to cover the entire plate. Bacterial lawns were grown at room temperature (22.5–24.5°) for 24 hr, and then stored at 4° until needed. All locomotion assays were performed on first-day adults at room temperature (22.5–24.5°). L4 stage larvae were picked the day before the assay and the experimenter was blind to the genotypes of the strains assayed. For experiments on strains carrying extrachromosomal arrays, the *sek-1*(*km4*) control worms were animals from the same plate that had lost the array.

Body bend assays were performed as described (Miller *et al.* 1999). A single animal was picked to the assay plate, the plate lid was returned, and the animal was allowed to recover for 30 sec. Body bends were then counted for 1 min, counting each time the worm’s tail reached the minimum or maximum amplitude of the sine wave. All strains in an experiment were assayed on the same assay plate. For experiments with *egl-8*, *unc-73*, and *rund-1* mutants, worms were allowed a minimal recovery period (until the worms started moving forward; 5 sec maximum) prior to counting body bends.

For the heat-shock experiment, plates of first-day adults were parafilmed and heat-shocked in a 34° water bath for 1 hr. Plates were then unparafilmed and incubated at 20° for 5 hr before performing body bend assays.

Radial locomotion assays were performed by picking animals to the middle of an assay plate. Assay plates were incubated at 20° for 20 hr and the distances of the worms from the starting point were measured.

Quantitative analysis of the waveform of worm tracks was performed as described (Topalidou *et al.* 2017). Briefly, worm tracks were photographed and ImageJ was used to measure the period and amplitude. The value for each animal was the average of five period-to-amplitude ratios.

### C. elegans pictures

Pictures of worms were taken at 60× magnification on a Nikon SMZ18 microscope with the DS-L3 camera control system. The worms were age-matched as first-day adults and each experiment set was photographed on the same locomotion assay plate prepared as described above. The images were processed using ImageJ and were rotated, cropped, and converted to grayscale.

### Molecular biology

Plasmids were constructed using the Gateway cloning system (Invitrogen). Plasmids and primers used are found in Table S2 in File S1. The *sek-1* cDNA was amplified by RT-PCR from worm RNA and cloned into a Gateway entry vector. To ensure proper expression of *sek-1*, an operon GFP was included in expression constructs with the following template: (promoter)p::*sek-1*(cDNA)::*tbb-2utr*::*gpd-2 operon*::*GFP*::*H2B:cye-1utr* (Frøkjær-Jensen *et al.* 2012). This resulted in untagged SEK-1, but expression could be monitored by GFP expression.

### Injections

*C. elegans* strains with extrachromosomal arrays were generated by standard methods (Mello *et al.* 1991). Injection mixes were made with a final total concentration of 100 ng/μl DNA. Constructs were injected at 5 ng/μl, injection markers at 5 ng/μl, and the carrier DNA Litmus 38i at 90 ng/μl. Multiple lines of animals carrying extrachromosomal arrays were isolated and had similar behaviors, as observed by eye. The line with the highest transmittance of the array was assayed.

### Statistical analyses

At the beginning of the project, a power study was conducted on pilot body bend assays using wild-type and *sek-1*(*yak42*) worms. To achieve a power of 0.95, it was calculated that 17 animals should be assayed per experiment. Data were analyzed to check for a normal distribution (using the D’Agostino–Pearson and Shapiro–Wilk normality tests), and then subjected to the appropriate analysis using GraphPad Prism 5. For data sets with three or more groups, if the data were normal, they were analyzed with a one-way ANOVA; if they were not, they were analyzed with a Kruskal–Wallis test. *Post hoc* tests were used to compare data sets within an experiment. Reported *P*-values are corrected. Table S3 in File S1 contains the statistical tests for each experiment. * *P* < 0.05, ** *P* < 0.01, *** *P* < 0.001.

### Data availability

Strains and plasmids are shown in Tables S1 and S2 in File S1, and are available from the *Caenorhabditis* Genetics Center or upon request. The authors state that all data necessary for confirming the conclusions presented in the article are represented fully within the article and Supplemental Material.

## Results

### sek-1 suppresses activated Gq

**Figure 1:**
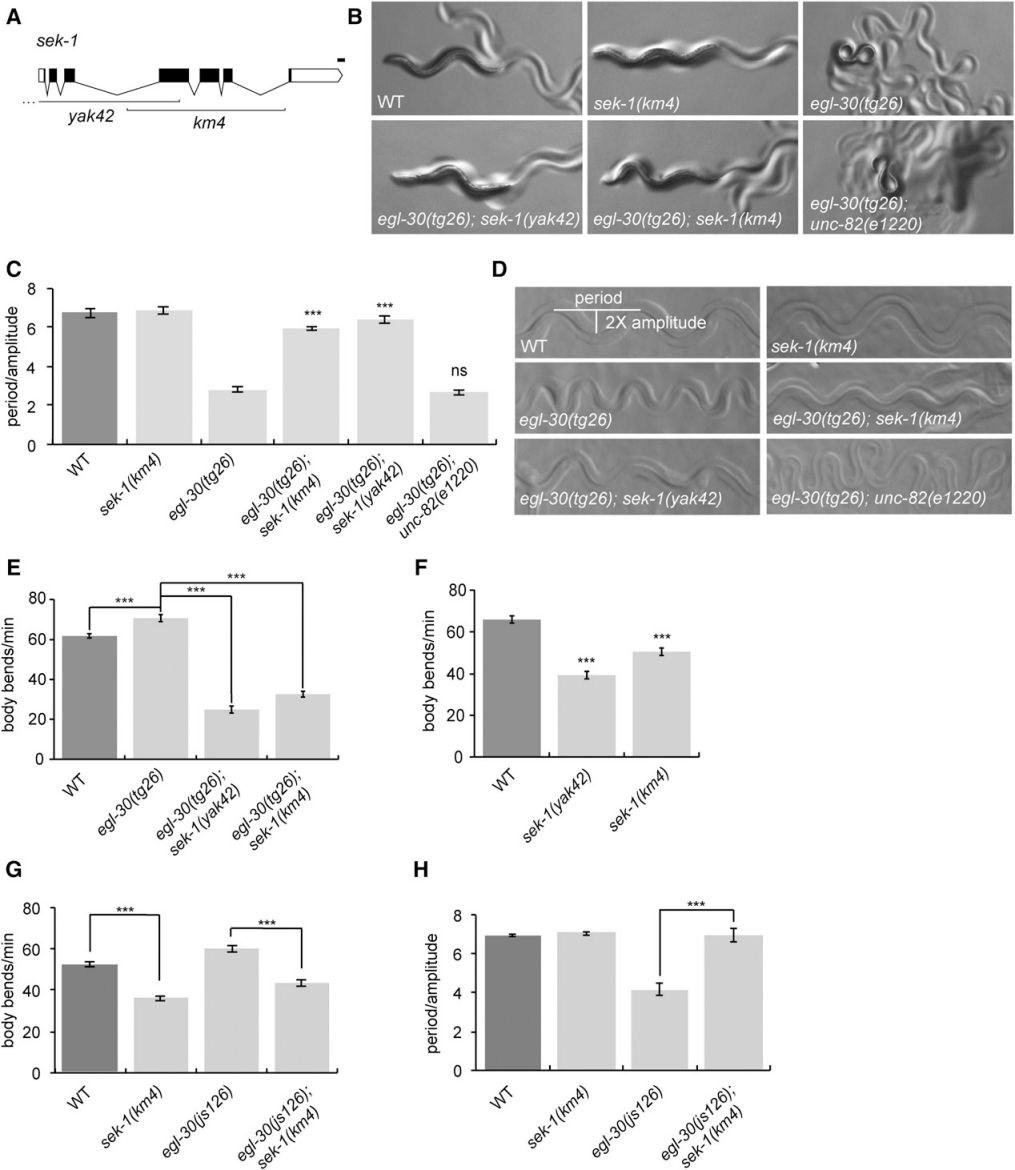
*sek-1* acts downstream of Gαq to modulate locomotion behavior. (A) Gene structure of *sek-1*. White boxes depict the 5′ and 3′ untranslated regions, black boxes depict exons, and lines show introns. The positions of the *yak42* and *km4* deletions are shown. *yak42* is a 3713-bp deletion that extends to 1926 bp upstream of the start codon. Drawn with Exon-Intron Graphic Maker (http://www.wormweb.org/exonintron). Bar, 100 bp. (B–D) *sek-1*(*yak42*) and *sek-1*(*km4*) suppress the loopy waveform of the activated Gq mutant *egl-30*(*tg26*). *unc-82*(*e1220*) does not suppress *egl-30*(*tg26*). (B) Photographs of first-day adult worms. All photos were taken at 60× magnification. (C) Quantification of the waveform phenotype. Error bars = SEM, *n* = 5. *** *P* < 0.001; ns, *P* > 0.05 compared to *egl-30*(*tg26*). (D) Photographs of worm tracks. (E) The activated Gq mutant *egl-30*(*tg26*) is hyperactive and is suppressed by *sek-1*(*yak42*) and *sek-1*(*km4*). Error bars = SEM, *n* = 20. *** *P* < 0.001. (F) *sek-1* mutant worms have slow locomotion. Error bars = SEM, *n* = 20. *** *P* < 0.001 compared to wild type. (G) *sek-1*(*km4*) suppresses the hyperactive locomotion of the activated Gq mutant *egl-30*(*js126*). Error bars = SEM, *n* = 20. *** *P* < 0.001. (H) *sek-1*(*km4*) suppresses the loopy waveform of the activated Gq mutant *egl-30*(*js126*). Error bars = SEM, *n* = 5. *** *P* < 0.001. WT, wild type.

To identify genes acting downstream of Gαq, we performed a forward genetic screen for suppressors of the activated Gq mutant, *egl-30*(*tg26*) (Doi and Iwasaki 2002). *egl-30*(*tg26*) worms are hyperactive and have a loopy posture, characterized by an exaggerated waveform (Figure 1, B–E). Thus, we screened for worms that were less hyperactive and less loopy. We isolated a recessive suppressor, *yak42*, and mapped it to the middle of the X chromosome (see *Materials and Methods*). Whole-genome sequencing revealed that *yak42* carries a large deletion of the *sek-1* gene from upstream of the start codon into exon 4 (Figure 1A). *yak42* also failed to complement *sek-1*(*km4*), a previously published *sek-1* deletion allele, for the Gq suppression phenotype (Figure 1A) (Tanaka-Hino *et al.* 2002).

*egl-30*(*tg26*) double mutants with either *sek-1*(*yak42*) or *sek-1*(*km4*) are not loopy (Figure 1, B–D) and are not hyperactive (Figure 1E and Figure S1A in File S1). *sek-1*(*yak42*) was outcrossed from *egl-30*(*tg26*) and assayed for locomotion defects. Both the *sek-1*(*yak42*) and *sek-1*(*km4*) mutants are coordinated but move more slowly than the wild type (Figure 1F). The *sek-1*(*ag1*) point mutation (Kim *et al.* 2002) also causes a similar slow locomotion phenotype (Figure S1B in File S1). To test whether the *egl-30*(*tg26*) suppression phenotype might be an indirect effect of the slow locomotion of a *sek-1* mutant, we built an *egl-30*(*tg26*) double mutant with a mutation in *unc-82*, a gene required for normal muscle structure. *unc-82* mutants are coordinated but move slowly, similar to *sek-1* mutants (Hoppe *et al.* 2010). However, although an *egl-30*(*tg26*) *unc-82*(*e1220*) double mutant moves more slowly than an *egl-30*(*tg26*) mutant (Figure S1C in File S1), it is still loopy (Figure 1, B–D). Thus, *sek-1* appears to be a specific suppressor of activated *egl-30*.

The *egl-30*(*tg26*) allele causes an R243Q missense mutation in the Gα switch III region that has been shown to reduce both the intrinsic GTPase activity of the G protein and render it insensitive to GTPase-activation by a regulator of G protein signaling (RGS) protein, thus leading to increased G protein activation (Natochin and Artemyev 2003). To test whether the suppression of *egl-30*(*tg26*) by *sek-1* is specific for this *egl-30* allele, we built a double mutant between *sek-1*(*km4*) and the weaker activating mutation *egl-30*(*js126*). *egl-30*(*js126*) causes a V180M missense mutation in the Gα switch I region immediately adjacent to one of the key residues required for GTPase catalysis (Hawasli *et al.* 2004). Thus, the *tg26* and *js126* alleles activate EGL-30 through different mechanisms. The *sek-1*(*km4*) mutant also suppresses the hyperactivity and loopy waveform of *egl-30*(*js126*) (Figure 1, G and H), demonstrating that *sek-1* suppression of activated *egl-30* is not allele specific.

EGL-30/Gαq is negatively regulated by GOA-1, the worm Gαo/i ortholog, and the RGS protein EAT-16 (Hajdu-Cronin *et al.* 1999). We tested whether *sek-1* also suppresses the *goa-1* and *eat-16* loss-of-functionmutants that cause a hyperactive and loopy phenotype similar to activated *egl-30* mutants. *sek-1*(*km4*) suppresses the hyperactivity and loopy waveform of *goa-1*(*sa734*) (Figure S1, D and E in File S1). However, although *sek-1*(*km4*) suppresses the hyperactivity of *eat-16*(*tm775*), it did not significantly suppress the loopy waveform (Figure S1, F and G in File S1). One possible downstream effector of GOA-1 is the DAG kinase DGK-1, which inhibits DAG-dependent functions such as synaptic vesicle release (Miller *et al.* 1999; Nurrish *et al.* 1999). *dgk-1*(*sy428*) animals are hyperactive, but the *sek-1dgk-1* double mutant is uncoordinated and looks like neither *sek-1* nor *dgk-1* mutants, confounding the interpretation of how *sek-1* genetically interacts with *dgk-1*.

### sek-1 acts in mature acetylcholine neurons

*egl-30* is widely expressed and acts in neurons to modulate locomotion (Lackner *et al.* 1999), so it is possible that *sek-1* also acts in neurons to modulate Gq signaling. *sek-1* is expressed in neurons, intestine, and several other tissues (Tanaka-Hino *et al.* 2002), and has been shown to function in GABA neurons to promote synaptic transmission (Vashlishan *et al.* 2008).

**Figure 2:**
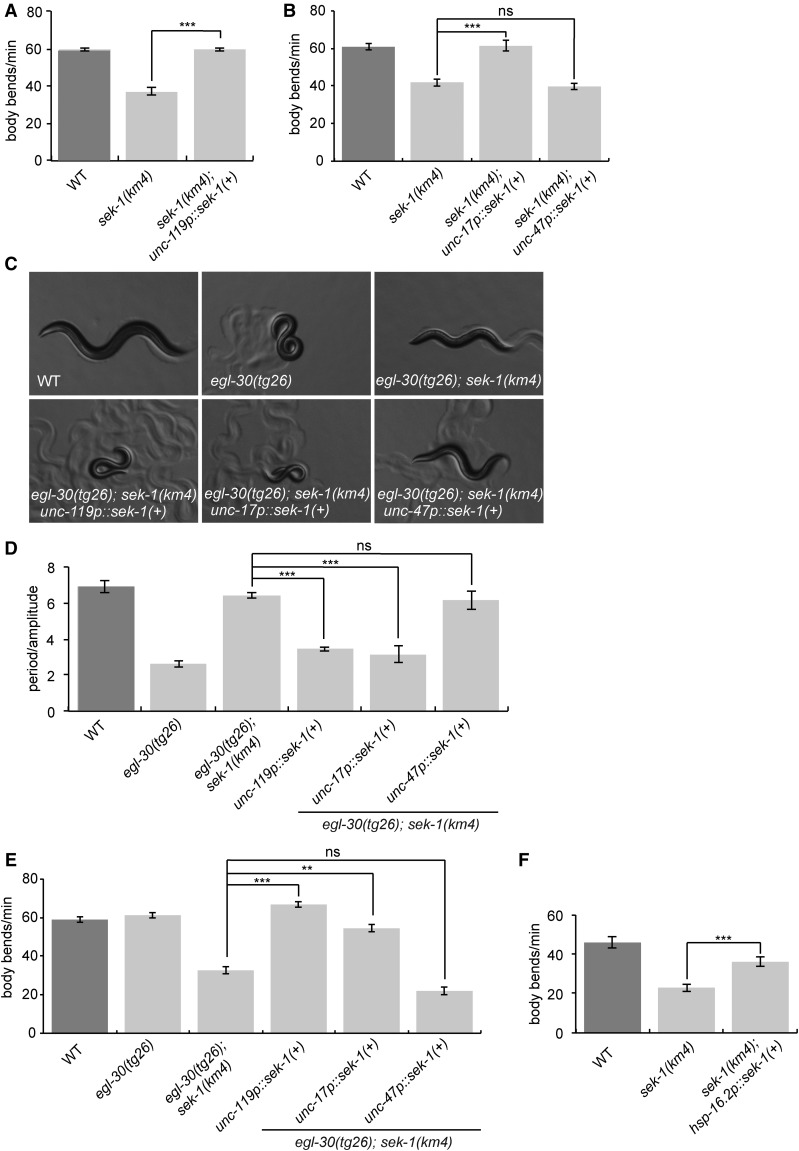
*sek-1* acts in mature acetylcholine neurons to modulate locomotion. (A) *sek-1* acts in neurons to modulate locomotion rate. The *sek-1* wild-type cDNA driven by the *unc-119* pan-neuronal promoter [*unc-119p*::*sek-1*(*+*)] rescues the slow locomotion phenotype of *sek-1*(*km4*) worms. Error bars = SEM, *n* = 20. *** *P* < 0.001. (B) *sek-1* acts in acetylcholine neurons to modulate locomotion rate. *sek-1* wild-type cDNA driven by the *unc-17* acetylcholine neuron promoter [*unc-17p*::*sek-1*(*+*)] rescues the slow locomotion phenotype of *sek-1*(*km4*) worms, but *sek-1* expression in GABA neurons using the *unc-47* promoter [*unc-47p*::*sek-1*(*+*)] does not. Error bars = SEM, *n* = 20. *** *P* < 0.001; ns, *P* > 0.05. (C and D) *sek-1* acts in acetylcholine neurons to modulate the loopy waveform of *egl-30*(*tg26*). *egl-30*(*tg26*) *sek-1*(*km4*) worms expressing *unc-119p*::*sek-1*(*+*) or *unc-17p*::*sek-1*(*+*) are loopy, similar to *egl-30*(*tg26*) worms, but *egl-30*(*tg26*) *sek-1*(*km4*) worms expressing *unc-47p*::*sek-1*(*+*) are similar to *egl-30*(*tg26*) *sek-1* worms. (C) Photographs of worms. All photos were taken at 60× magnification. (D) Quantification of waveform phenotype. Error bars = SEM, *n* = 5. *** *P* < 0.001; ns, *P* > 0.05. (E) *sek-1* acts in acetylcholine neurons to modulate the locomotion rate of *egl-30*(*tg26*). *egl-30*(*tg26*) *sek-1*(*km4*) worms expressing *unc-119p*::*sek-1*(*+*) or *unc-17p*::*sek-1*(*+*) have an increased locomotion rate compared to *egl-30*(*tg26*) *sek-1*, but *egl-30*(*tg26*) *sek-1*(*km4*) worms expressing *unc-47p*::*sek-1*(*+*) are similar to *egl-30*(*tg26*) *sek-1*. Error bars = SEM, *n* = 17–20. *** *P* < 0.001, ** *P* < 0.01; ns, *P* > 0.05. (F) *sek-1* acts in mature neurons to modulate locomotion rate. Heat-shock induced expression of *sek-1* in adults [*hsp-16.2p*::*sek-1*(*+*)] rescues the slow locomotion phenotype of *sek-1*(*km4*). Error bars = SEM, *n* = 20. *** *P* < 0.001. WT, wild type.

To identify the cell type responsible for the *sek-1* locomotion phenotypes, we expressed the wild-type *sek-1* cDNA under different cell-specific promoters and tested for transgenic rescue of a *sek-1* null mutant. Expression of *sek-1* in all neurons (using the *unc-119* promoter) or in acetylcholine neurons (*unc-17* promoter) was sufficient to rescue the *sek-1* mutant slow locomotion phenotype, but expression in GABA neurons (*unc-47* promoter) was not sufficient (Figure 2, A and B). These results indicate that *sek-1* acts in acetylcholine neurons to modulate locomotion rate.

We next tested whether *sek-1* acts in neurons to suppress *egl-30*(*tg26*). Expression of *sek-1* under pan-neuronal and acetylcholine neuron promoters reversed the *sek-1* suppression of *egl-30*(*tg26*). Specifically, *egl-30*(*tg26*) *sek-1* double mutants expressing wild-type *sek-1* in all neurons or acetylcholine neurons resembled the *egl-30*(*tg26*) single mutant (Figure 2, C–E). However, expression of *sek-1* in GABA neurons did not reverse the suppression phenotype (Figure 2, C–E). Together, these data show that *sek-1* acts in acetylcholine and not GABA neurons to modulate both wild-type locomotion rate and Gq signaling.

To narrow down the site of *sek-1* action, we expressed *sek-1* in head (*unc-17H* promoter) and motorneuron (*unc-17β* promoter) acetylcholine neuron subclasses (Topalidou *et al.* 2017). Expression of *sek-1* in acetylcholine motorneurons rescued the *sek-1* slow locomotion phenotype (Figure S2A in File S1), suggesting that the slow locomotion of *sek-1* mutants is due to a loss of *sek-1* in acetylcholine motorneurons. However, expression of *sek-1* in either the head acetylcholine neurons or motorneurons partially reversed the *sek-1* suppression of *egl-30*(*tg26*) hyperactivity (Figure S2B in File S1), suggesting that the hyperactivity of activated Gq mutants may result from excessive Gq signaling in both head acetylcholine neurons and acetylcholine motorneurons; *sek-1* may act in Gq signaling in both neuronal cell types. By contrast, expression of *sek-1* in head acetylcholine neurons but not motorneurons reversed the *sek-1* suppression of the *egl-30*(*tg26*) loopy waveform (Figure S2C in File S1), suggesting that the loopy posture of activated Gq mutants may result from excessive Gq signaling in head acetylcholine neurons, and *sek-1* may act in those neurons to control body posture.

Because *sek-1* acts in the development of the AWC asymmetric neurons, we asked whether *sek-1* also has a developmental role in modulating locomotion by testing whether adult-specific *sek-1* expression (driven by a heat-shock promoter) is sufficient to rescue the *sek-1* mutant. We found that *sek-1* expression in adults rescues the *sek-1* slow locomotion phenotype (Figure 2F). This result indicates that *sek-1* is not required for development of the locomotion circuit, and instead acts in mature neurons to modulate locomotion.

### The p38 MAPK pathway is a positive regulator of Gq signaling

**Figure 3:**
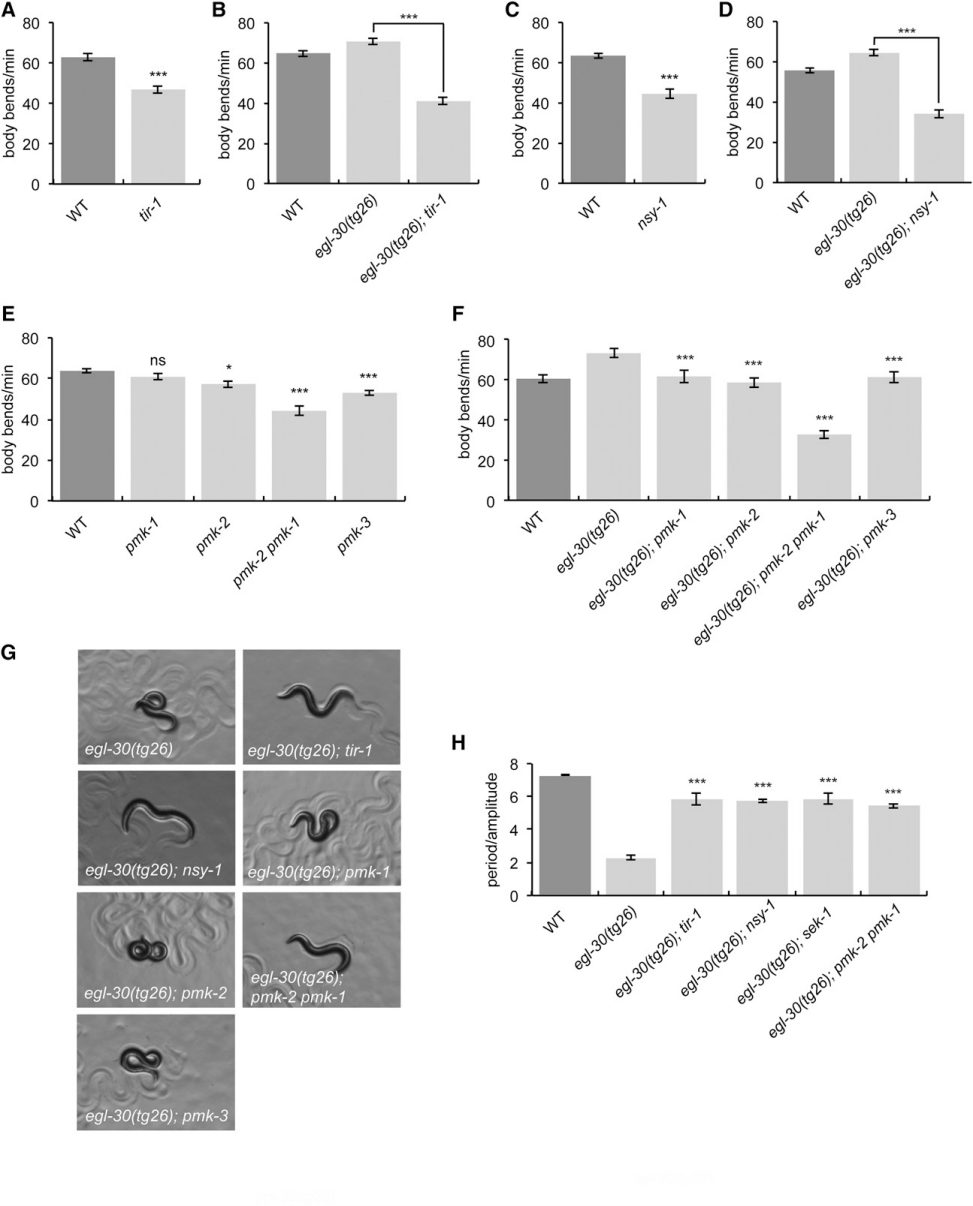
The p38 MAPK pathway modulates locomotion downstream of *egl-30*. (A) *tir-1*(*tm3036*) mutant animals have slow locomotion. Error bars = SEM, *n* = 20. *** *P* < 0.001. (B) *tir-1*(*tm3036*) suppresses *egl-30*(*tg26*). *egl-30*(*tg26*) *tir-1* animals move more slowly than the hyperactive *egl-30*(*tg26*) animals. Error bars = SEM, *n* = 20. *** *P* < 0.001. (C) *nsy-1*(*ok593*) mutant animals have slow locomotion. Error bars = SEM, *n* = 20. *** *P* < 0.001. (D) *nsy-1*(*ok593*) suppresses *egl-30*(*tg26*). *egl-30*(*tg26*) *nsy-1* animals move more slowly than hyperactive *egl-30*(*tg26*) animals. Error bars = SEM, *n* = 20. *** *P* < 0.001. (E) *pmk-2*, *pmk-2 pmk-1*, and *pmk-3* mutant animals have slow locomotion. Error bars = SEM, *n* = 20. * *P* < 0.05, *** *P* < 0.001, and ns, *P* > 0.05 compared to wild type. (F) A *pmk-2 pmk-1* double mutant suppresses the hyperactivity of *egl-30*(*tg26*). Error bars = SEM, *n* = 20. *** *P* < 0.001 compared to *egl-30*(*tg26*). (G and H) *tir-1*(*tm3036*), *nsy-1*(*ok593*), and the *pmk-2 pmk-1* double mutant suppress the loopy waveform of *egl-30*(*tg26*). *egl-30*(*tg26*) animals with mutations in either *pmk-1*, *pmk-2*, or *pmk-3* are still loopy. (G) Worm photographs. All photos were taken at 60X magnification. (H) Quantification. Error bars = SEM, *n* = 5. *** *P* < 0.001 compared to *egl-30*(*tg26*). WT, wild type.

SEK-1 is the MAPKK in the p38 MAPK pathway consisting of the adaptor protein TIR-1, NSY-1 (MAPKKK), SEK-1 (MAPKK), and PMK-1 or PMK-2 (MAPKs) (Tanaka-Hino *et al.* 2002; Andrusiak and Jin 2016). We tested whether the entire p38 MAPK signaling module also modulates locomotion rate and suppression of activated Gq. Both *tir-1*(*tm3036*) and *nsy-1*(*ok593*) mutant animals have slow locomotion on their own, and also suppress the hyperactivity and loopy waveform of *egl-30*(*tg26*) (Figure 3, A–D, G, and H). We also tested single mutants in each of the three worm p38 MAPK genes (*pmk-1*, *pmk-2*, and *pmk-3*) and a *pmk-2pmk-1* double mutant. Although we found that the *pmk-2* and *pmk-3* single mutants were slightly slow on their own, only the *pmk-2pmk-1* double mutant phenocopied *sek-1* and suppressed both the hyperactivity and loopy waveform of *egl-30*(*tg26*) (Figure 3, E–H). Thus, *pmk-2* and *pmk-1* act redundantly downstream of *sek-1* to suppress *egl-30*(*tg26*). These data suggest that the p38 MAPK pathway modulates locomotion rate in *C. elegans* and acts genetically downstream of *egl-30*.

The JNK MAPK pathway, related to the p38 MAPK family, also modulates locomotion in *C. elegans*. Specifically, the JNK pathway members *jkk-1* (JNK MAPKK) and *jnk-1* (JNK MAPK) have been shown to act in GABA neurons to modulate locomotion (Kawasaki 1999). We found that the *jkk-1* and *jnk-1* single mutants had slow locomotion and that the double mutants with p38 MAPK pathway members exhibited an additive slow locomotion phenotype (Figure S3A in File S1). Moreover, neither *jkk-1* nor *jnk-1* suppressed the loopy phenotype of *egl-30*(*tg26*) (Figure S3B in File S1). Thus, the JNK and p38 MAPK pathways modulate locomotion independently, and the JNK pathway is not involved in Gq signaling.

We also tested the involvement of possible p38 MAPK pathway effectors. One of the targets of PMK-1 is the transcription factor ATF-7 (Shivers *et al.* 2010). Both the *atf-7*(*qd22qd130*) loss-of-function mutant and the *atf-7*(*qd22*) gain-of-function mutant moved slowly compared to wild-type animals (Figure S3C in File S1). However, *atf-7*(*qd22qd130*) did not suppress the loopy waveform of *egl-30*(*tg26*) (Figure S3B in File S1), suggesting that *atf-7* is not a target of this pathway, or else it acts redundantly with other downstream p38 MAPK targets. We also tested *gap-2*, the closest *C. elegans* homolog of ASK1-interacting protein (AIP1), which activates ASK1 (the ortholog of *C. elegans* NSY-1) in mammalian systems (Zhang *et al.* 2003). A *C. elegans gap-2* mutant showed no locomotion defect (Figure S3D in File S1). Finally, we tested VHP-1, a phosphatase for p38 and JNK MAPKs that inhibits p38 MAPK signaling (Kim *et al.* 2004). However, the *vhp-1*(*sa366*) mutant also showed no locomotion defect (Figure S3D in File S1).

*egl-30*(*tg26*) animals are loopy and hyperactive, so we tested whether increased activation of the TIR-1/p38 MAPK signaling module causes similar phenotypes. The *tir-1*(*ky648*) allele leads to a gain-of-function phenotype in the AWC neuron specification (Chang *et al.* 2011), but does not cause loopy or hyperactive locomotion (Figure S3, E and F in File S1).

### Genetic interactions of sek-1 with pathways acting downstream of Gq

Our forward genetic screen for suppressors of *egl-30*(*tg26*) identified mutants that fall into three different categories: mutants in the canonical Gq pathway, such as the PLC *egl-8* ((Lackner *et al.* 1999); mutants in the RhoGEF Trio pathway, such as *unc-73* (Williams *et al.* 2007); and mutants that affect dense-core vesicle biogenesis and release (Ailion *et al.* 2014; Topalidou *et al.* 2016).

**Figure 4:**
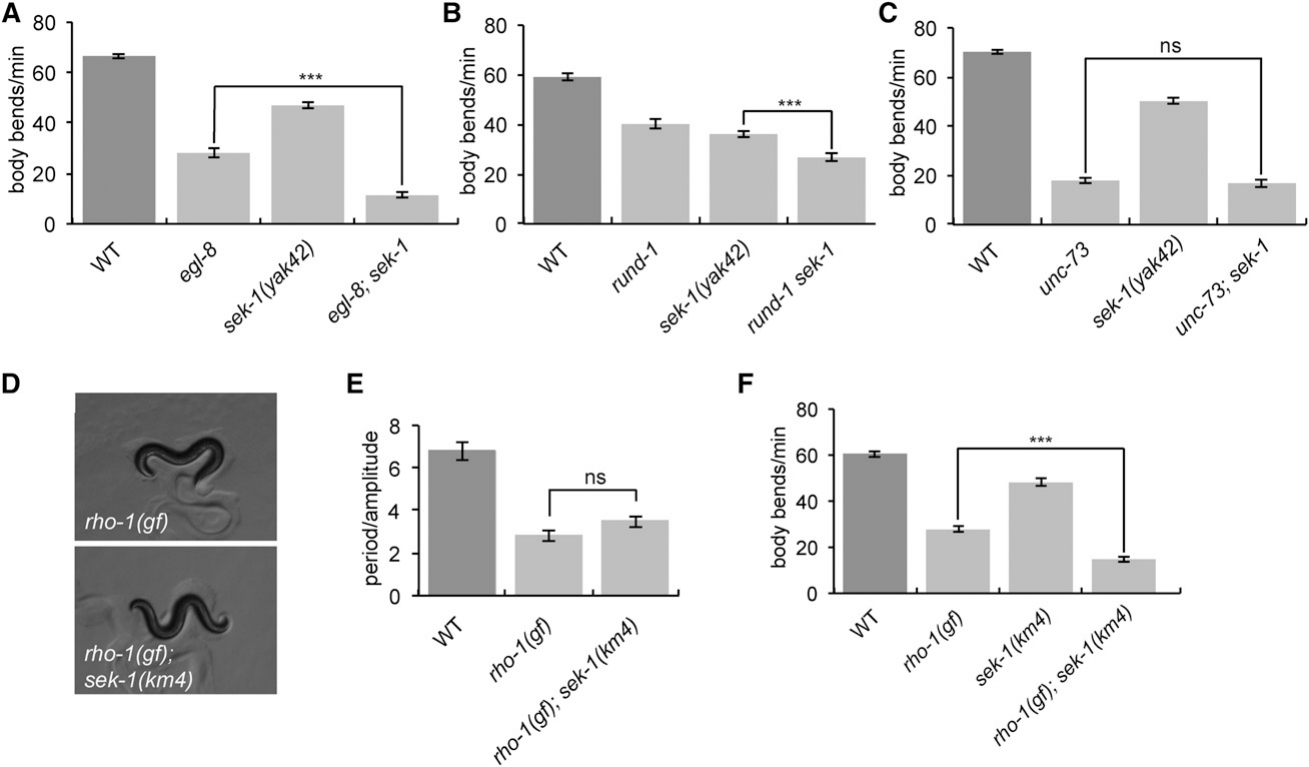
*sek-1* acts in the same genetic pathway as *unc-73*. (A) *sek-1* does not act in the same genetic pathway as *egl-8*. The *sek-1*(*yak42*) mutation enhances the slow locomotion of the *egl-8*(*sa47*) mutant. Error bars = SEM, *n* = 20. *** *P* < 0.001. (B) *sek-1* does not act in the same genetic pathway as *rund-1*. The *sek-1*(*yak42*) mutation enhances the slow locomotion of the *rund-1*(*tm3622*) mutant. Error bars = SEM, *n* = 20. *** *P* < 0.001. (C) *sek-1* may act in the same genetic pathway as *unc-73*. The *sek-1*(*yak42*) mutation does not enhance the slow locomotion phenotype of the *unc-73*(*ox317*) mutant. Error bars = SEM, *n* = 20. ns, *P* > 0.05. (D and E) *sek-1*(*km4*) does not suppress the loopy waveform of *nzIs29* [*rho-1*(*gf*)] animals. (D) Worm photographs. Photos were taken at 60× magnification. (E) Quantification. Error bars = SEM, *n* = 5. ns, *P* > 0.05. (F) *sek-1*(*km4*) does not suppress the slow locomotion of *rho-1*(*gf*) animals. Error bars = SEM, *n* = 20. *** *P* < 0.001. WT, wild type.

To test if *sek-1* acts in any of these pathways, we built double mutants between *sek-1* and members of each pathway. Loss-of-function alleles of *egl-8*(*sa47*), *unc-73*(*ox317*), and *rund-1*(*tm3622*) have slow locomotion (Figure 4, A–C). We found that *sek-1* enhances the slow locomotion phenotype of *egl-8* and *rund-1* single mutants, suggesting that *sek-1* does not act in the same pathway as *egl-8* or *rund-1* (Figure 4, A and B). By contrast, *sek-1* does not enhance the slow locomotion phenotype of *unc-73* mutants (Figure 4C), suggesting that *sek-1* may act in the same genetic pathway as the Trio RhoGEF *unc-73*.

We next tested whether *sek-1* interacts with *rho-1*, encoding the small G protein Rho that is activated by Trio. Because *rho-1* is required for viability (Jantsch-Plunger *et al.* 2000), we used an integrated transgene overexpressing an activated *rho-1* mutant allele specifically in acetylcholine neurons. Animals carrying this activated RHO-1 transgene, referred to here as *rho-1*(*gf*), have a loopy posture reminiscent of *egl-30*(*tg26*) (McMullan *et al.* 2006), and a decreased locomotion rate (Figure 4, D–F). *rho-1*(*gf*) *sek-1*(*km4*) double mutants had a loopy body posture like *rho-1*(*gf*) animals, and an even slower locomotion rate (Figure 4, D–F), suggesting that *sek-1* and *rho-1*(*gf*) mutants have additive locomotion phenotypes. However, both *sek-1*(*km4*) and *sek-1*(*yak42*) weakly suppress the slow growth rate of the *rho-1*(*gf*) mutant (data not shown). Because *sek-1* does not enhance *unc-73* mutants and suppresses some aspects of the *rho-1*(*gf*) mutant, *sek-1* may modulate output of the Rho pathway, although it probably is not a direct transducer of Rho signaling.

### sek-1 and nsy-1 partially suppress activated NCA

To clarify the relationship of the SEK-1 p38 MAPK pathway to the Rho pathway acting downstream of Gq, we examined interactions with *nca-1*, a downstream target of the Gq-Rho pathway (Topalidou *et al.* 2017). NCA-1 and its orthologs are sodium leak channels associated with rhythmic behaviors in several organisms (Nash *et al.* 2002; Lu *et al.* 2007; Shi *et al.* 2016). In *C. elegans*, NCA-1 potentiates persistent motor circuit activity and sustains locomotion (Gao *et al.* 2015).

**Figure 5:**
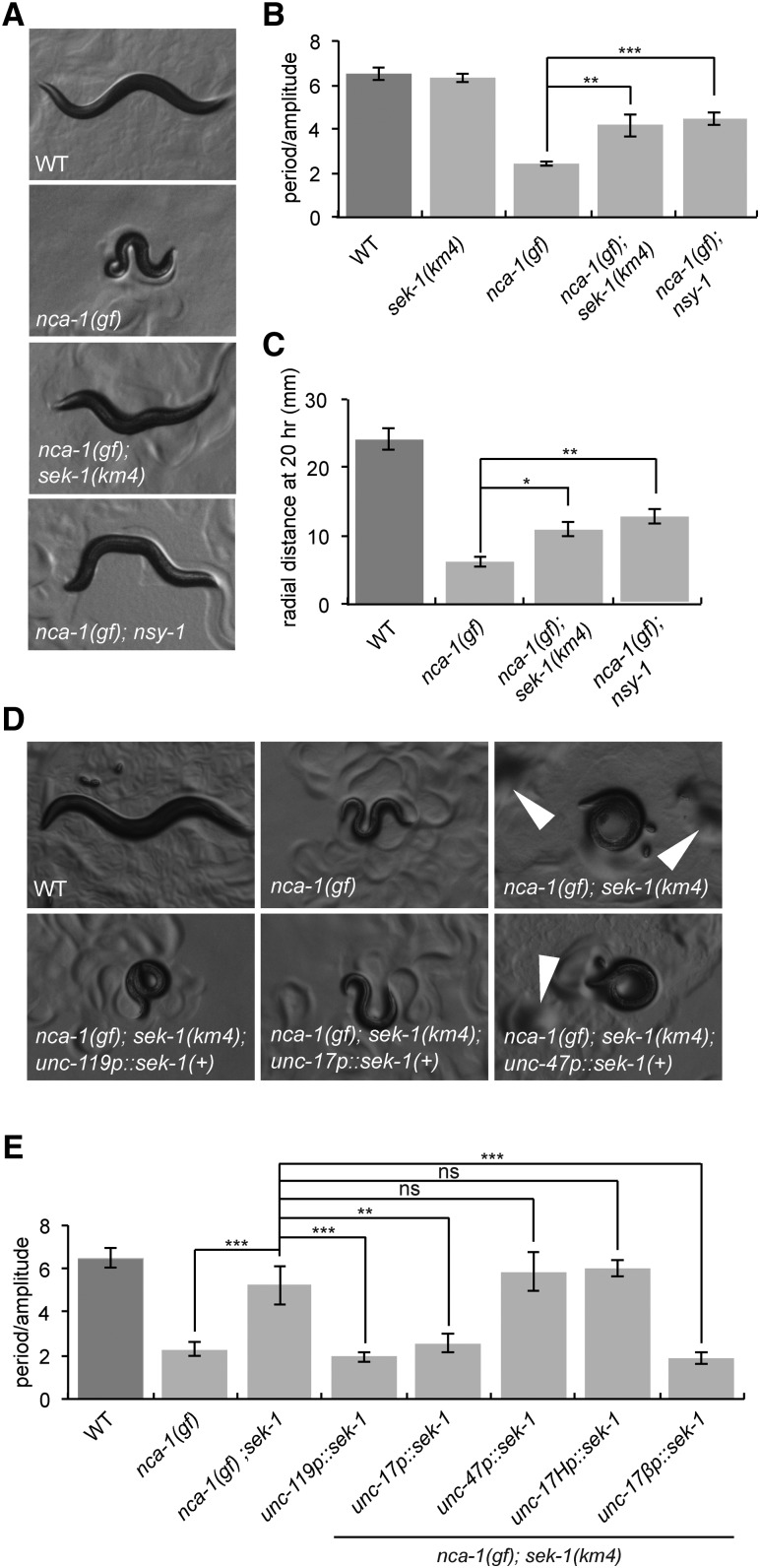
*sek-1* and *nsy-1* weakly suppress *nca-1*(*gf*). (A) *nca-1*(*gf*) mutants are small, loopy, and uncoordinated. The phenotypes of *nca-1*(*ox352*) animals are partially suppressed by *sek-1*(*km4*) and *nsy-1*(*ok593*). Photographs of first-day adults. All photos were taken at 60× magnification. (B) *sek-1*(*km4*) and *nsy-1*(*ok593*) suppress the loopy waveform of *nca-1*(*gf*). Error bars = SEM, *n* = 5. ** *P* < 0.01, *** *P* < 0.001. (C) *sek-1* and *nsy-1* suppress *nca-1*(*gf*) locomotion. *nca-1*(*gf*) animals travel a small distance from the center of the plate in the radial locomotion assay. *nca-1*(*gf*) *nsy-1*(*ok593*) and *nca-1*(*gf*) *sek-1*(*km4*) worms move further than *nca-1*(*gf*) worms. Error bars = SEM, *n* = 30. * *P* < 0.05, ** *P* < 0.01. (D) *sek-1* acts in acetylcholine neurons to modulate the size and loopy waveform of *nca-1*(*gf*). *nca-1*(*ox352*) *sek-1*(*km4*) animals expressing *sek-1* in all neurons [*unc-119p*::*sek-1*(*+*)] or in acetylcholine neurons [*unc-17p*::*sek-1*(*+*)] are loopy and small like *nca-1*(*gf*) animals, but *nca-1*(*ox352*) *sek-1*(*km4*) animals expressing *sek-1* in GABA neurons [*unc-47p*::*sek-1*(*+*)] resemble *nca-1*(*gf*) *sek-1* animals. White arrowheads depict food piles created by *nca-1*(*gf*) *sek-1*(*km4*) animals due to their uncoordinated locomotion. Such food piles are not made by *nca-1*(*gf*) animals. (E) *sek-1* acts in acetylcholine motorneurons to modulate the loopy waveform of *nca-1*(*gf*). *nca-1*(*ox352*) *sek-1*(*km4*) worms expressing *sek-1* in all neurons [*unc-119p*::*sek-1*(*+*)], acetylcholine neurons [*unc-17p*::*sek-1*(*+*)], or acetylcholine motorneurons [*unc-17βp*::*sek-1*(*+*)] are loopy like *nca-1*(*gf*), but *nca-1*(*ox352*) *sek-1*(*km4*) worms expressing *sek-1* in GABA neurons [*unc-47p*::*sek-1*(*+*)] or head acetylcholine neurons [*unc-17Hp*::*sek-1*(*+*)] are similar to *nca-1*(*gf*) *sek-1*. Error bars = SEM, *n* = 5. ** *P* < 0.01, *** *P* < 0.001; ns, *P* > 0.05. WT, wild type.

We tested whether *sek-1* and *nsy-1* mutants suppress the activated NCA-1 mutant *ox352*, referred to as *nca-1*(*gf*). The *nca-1*(*gf*) animals are coiled and uncoordinated; thus, it is difficult to measure their locomotion rate by the body bend assay because they do not reliably propagate sinusoidal waves down the entire length of their body. Instead, we used a radial locomotion assay in which we measured the distance animals moved from the center of a plate. *nca-1*(*gf*) double mutants with either *sek-1*(*km4*) or *nsy-1*(*ok593*) uncoiled a bit, but still exhibited uncoordinated locomotion (Figure 5A). In fact, although these double mutants showed more movement in the anterior half of their bodies than *nca-1*(*gf*), they propagated body waves to their posterior half even more poorly than the *nca-1*(*gf*) mutant. However, both *sek-1* and *nsy-1* partially suppressed the loopy waveform of the *nca-1*(*gf*) mutant (Figure 5, A and B), and in radial locomotion assays, *sek-1* and *nsy-1* weakly suppressed the *nca-1*(*gf*) locomotion defect (Figure 5C). Additionally, both *sek-1* and *nsy-1* partially suppressed the small body size of *nca-1*(*gf*) (Figure S4A in File S1). Together, these data suggest that mutations in the SEK-1 p38 MAPK pathway suppress some aspects of the *nca-1*(*gf*) mutant.

Given that *sek-1* acts in acetylcholine neurons to modulate wild-type and *egl-30*(*tg26*) locomotion, we tested whether *sek-1* also acts in these neurons to suppress *nca-1*(*gf*). Expression of *sek-1* in all neurons or in acetylcholine neurons of *nca-1*(*gf*) *sek-1*(*km4*) animals restored the *nca-1*(*gf*) loopy phenotype (Figure 5, D and E). By contrast, expression of *sek-1* in GABA neurons did not affect the loopy posture of the *nca-1*(*gf*) *sek-1* double mutant (Figure 5, D and E). These data suggest that *sek-1* acts in acetylcholine neurons to modulate the body posture of *nca-1*(*gf*) as well. However, in radial locomotion assays, expression of *sek-1* in any of these neuron classes did not significantly alter the movement of the *nca-1*(*gf*) *sek-1* double mutant (Figure S4B in File S1), although the weak suppression of *nca-1*(*gf*) by *sek-1* in this assay makes it difficult to interpret these negative results. To further narrow down the site of action of *sek-1* for its NCA suppression phenotypes, we expressed it in subclasses of acetylcholine neurons. Surprisingly, expression of *sek-1* in acetylcholine motorneurons but not head acetylcholine neurons was sufficient to restore the loopy posture of the *nca-1*(*gf*) mutant (Figure 5E), the opposite of what we found for *sek-1* modulation of the loopy posture of the activated Gq mutant, suggesting that the loopy posture of *nca-1*(*gf*) mutants may result from excessive NCA-1 activity in acetylcholine motorneurons. Additionally, expression of *sek-1* in either the head acetylcholine neurons or the motorneurons restored the *nca-1*(*gf*) small body size phenotype (Figure S4C in File S1). We make the tentative conclusion that *sek-1* acts in acetylcholine neurons to modulate *nca-1*(*gf*) body posture and size, but we are not able to conclusively narrow down its site of action further, possibly due to the uncoordinated phenotype of *nca-1*(*gf*) and the weaker suppression of *nca-1*(*gf*) by *sek-1*.

## Discussion

The p38 MAPK pathway has been best characterized as a pathway activated by a variety of cellular stresses and inflammatory cytokines (Kyriakis and Avruch 2012), but it has also been implicated in neuronal function, including some forms of mammalian synaptic plasticity (Bolshakov *et al.* 2000; Rush *et al.* 2002; Huang *et al.* 2004). In this study, we identified a new neuronal role for the MAPKK SEK-1 and the p38 MAPK pathway as a positive modulator of locomotion rate and Gq signaling. The physiological importance of this pathway is clear under conditions of elevated Gq signaling but is less obvious during normal wild-type locomotion, consistent with the observation that *sek-1* mutations have a relatively weak effect on synaptic transmission in a wild-type background (Vashlishan *et al.* 2008). Thus, the SEK-1 p38 MAPK pathway may be more important for modulation of Gq signaling and synaptic strength than for synaptic transmission *per se*.

In addition to SEK-1, we identified other p38 pathway components that modulate Gq signaling. Specifically, we found that *tir-1*, *nsy-1*, and *pmk-1pmk-2* mutants exhibit locomotion defects identical to *sek-1* and suppress activated Gq, suggesting that they act in a single p38 pathway to modulate signaling downstream of Gq. These results indicate a redundant function for PMK-1 and PMK-2 in modulating locomotion rate and Gq signaling. PMK-1 and PMK-2 also act redundantly for some other neuronal roles of the p38 pathway, such as the development of the asymmetric AWC neurons and to regulate induction of serotonin biosynthesis in the ADF neurons in response to pathogenic bacteria (Shivers *et al.* 2009; Pagano *et al.* 2015). By contrast, PMK-1 acts alone in the intestine to regulate innate immunity, and in interneurons to regulate trafficking of the GLR-1 glutamate receptor (Pagano *et al.* 2015; Park and Rongo 2016).

What are the downstream effectors of the SEK-1 p38 MAPK pathway that modulate locomotion? There are several known downstream effectors of p38 MAPK signaling in *C. elegans*, including the transcription factor ATF-7 (Shivers *et al.* 2010). Our data indicate that ATF-7 is not required for the p38 MAPK–dependent modulation of Gq signaling. The p38 MAPK pathway may activate molecules other than transcription factors, or may activate multiple downstream effectors.

How does the SEK-1 p38 pathway modulate the output of Gq signaling? One of the pathways that transduces signals from Gq includes the RhoGEF Trio/UNC-73, the small GTPase Rho, and the cation channel NALCN/NCA-1 (Williams *et al.* 2007; Topalidou *et al.* 2017). Compared to other pathways downstream of Gq, mutants in the Rho-Nca pathway are particularly strong suppressors of the loopy waveform phenotype of the activated Gq mutant (Topalidou *et al.* 2017). Similarly, we found that mutations in the SEK-1 p38 MAPK pathway strongly suppress the loopy waveform of the activated Gq mutant, suggesting that the SEK-1 pathway might modulate Gq signal output through the Rho-Nca branch. Consistent with this, we found that mutations in the SEK-1 p38 MAPK pathway partially suppress an activated NCA-1 mutant. Given the precedence for direct phosphorylation of sodium channels by p38 to regulate channel properties (Wittmack *et al.* 2005; Hudmon *et al.* 2008), it is possible that PMK-1 and PMK-2 phosphorylate NCA-1 to regulate its expression, localization, or activity.

Consistent with the observation that Gq acts in acetylcholine neurons to stimulate synaptic transmission (Lackner *et al.* 1999), we found that *sek-1* acts in acetylcholine neurons to modulate the locomotion rate in both wild-type and activated Gq mutants. *sek-1* also acts in acetylcholine neurons to modulate the loopy waveform of both activated Gq and activated *nca-1* mutants, and the size of activated *nca-1* mutants. However, our data obtained from attempting to narrow down the site of action of *sek-1* suggest that it may act in both head acetylcholine neurons and acetylcholine motorneurons, and that the waveform is probably controlled by at least partially distinct neurons from those that control locomotion rate. Further work will be required to identify the specific neurons where Gq, NCA-1, and the SEK-1 pathway act to modulate locomotion rate and waveform, and determine whether they all act together in the same cell.

## Supplementary Material

Supplemental material is available online at www.g3journal.org/lookup/suppl/doi:10.1534/g3.117.043273/-/DC1.
