# Supplementary material for: The SEK-1 p38 MAP Kinase Pathway Modulates Gq Signaling in Caenorhabditis elegans
Source: G3 (Bethesda). 2017 Jul 7;7(9):2979–89. doi: 10.1534/g3.117.043273 (PMC5592925; doi:10.1534/g3.117.043273)
Supplement: Supplementary file 1 [file 2979FileS1.pdf]

Supplementary Information

Table S1. Strain List

Strain	Genotype
AU1	*sek-1(ag1)* X
BS3383	*pmk-3(ok169)* IV
CX3695	*kyIs140[str-2p::gfp, lin-15(+)]* I
CX5959	*kyIs140[str-2p::gfp, lin-15(+)]* I; *tir-1(ky648gf)* III
EG317	*unc-73(ox317)* I
EG1000	*dpy-5(e61)* I; *rol-6(e187)* II; *lon-1(e1820)* III
EG1020	*bli-6(sc16)* IV; *dpy-11(e224)* V; *lon-2(e678)* X
EG3745	*eat-16(tm775)* I *; him-5(e1490)* V
EG4782	*nzIs29[unc-17p::rho-1(G14V), unc-122::gfp]* II
EG5505	*rund-1(tm3622)* X
EG7989	*unc-119(ed3)* III; *oxTi668[eft-3p::TdTomato::H2B, Cb-unc-119(+)]* X
IG685	*tir-1(tm3036)* III
JN147	*gap-2(tm748)* X
JT47	*egl-8(sa47)* V
JT366	*vhp-1(sa366)* II
JT734	*goa-1(sa734)* I
KU2	*jkk-1(km2)* X
KU4	*sek-1(km4)* X
KU25	*pmk-1(km25)* IV
N2	Bristol wild isolate, standard lab wild-type
NM1380	*egl-30(js126gf)* I

VC8	*jnk-1(gk7)* IV
VC390	*nsy-1(ok593)* IV
XZ42	*sek-1(yak42)* X
XZ1233	*egl-30(tg26)* I*; sek-1(yak42)* X
XZ1151	*egl-30(tg26)* I
XZ1566	*egl-8(sa47)* V*; sek-1(yak42)* X
XZ1567	*unc-73(ox317)* I*; sek-1(yak42)* X
XZ1574	*rund-1(tm3622) sek-1(yak42)* X
XZ1575	*egl-30(tg26)* I*; sek-1(km4)* X
XZ1588	*egl-30(tg26)* I*; nsy-1(ok593)* IV
XZ1589	*egl-30(tg26)* I*; sek-1(km4)* X*; qdEx8[unc-119p::sek-1::GFP, myo-2p::mStrawberry::unc-54-3'UTR]*
XZ1590	*egl-30(tg26)* I *; jkk-1(km2)* X
XZ1593	*egl-30(tg26)* I*; pmk-1(km25)* IV
XZ1597	*egl-30(tg26)* I *; jnk-1(gk7)* IV
XZ1642	*sek-1(km4)* X*; yakEx72[unc-17p::sek-1::tbb-2utr-operon-GFP::H2B::cye-1utr, myo-2p::mCherry]*
XZ1643	*sek-1(km4)* X*; yakEx73[unc-47p::sek-1::tbb-2utr-operon-GFP::H2B::cye-1utr, myo-2p::mCherry]*
XZ1717	*nzIs29[unc-17p::rho-1(G14V) unc-122::gfp]* II*; sek-1(km4)* X
XZ1720	*sek-1(km4)* X *; yakEx82[unc-17Hp::sek-1:: tbb-2utr-operon-GFP::H2B::cye-1utr, myo-2p::mCherry]*
XZ1721	*sek-1(km4)* X *; yakEx83[unc-17βp::sek-1::tbb-2utr-operon-GFP::H2B::cye-1utr, myo-2p::mCherry]*
XZ1770	*egl-30(tg26)* I*; pmk-2(qd279 qd171) pmk-1(km25)* IV

XZ1771	*egl-30(tg26)* I*; pmk-2(qd287)* IV
XZ1772	*egl-30(tg26)* I*; pmk-3(ok169)* IV
XZ1815	*egl-30(tg26)* I*; tir-1(tm3036)* III
XZ1816	*nca-1(ox352)* IV*; sek-1(km4)* X*; qdEx8[unc-119p::sek-1::GFP::unc-54-3' UTR, myo-2p::mStrawberry::unc-54-3'UTR]*
XZ1820	*nca-1(ox352)* IV *; sek-1(km4)* X *; yakEx83[unc-17βp::sek-1::tbb-2utr-operon-GFP::H2B::cye-1utr, myo-2p::mCherry]*
XZ1830	*egl-30(tg26)* I *; sek-1(km4)* X *; yakEx83[unc-17βp::sek-1::tbb-2utr-operon-GFP::H2B::cye-1utr, myo-2p::mCherry]*
XZ1834	*egl-30(tg26)* I*; sek-1(km4)* X*; yakEx72[unc-17p::sek-1::tbb-2utr-operon-GFP::H2B::cye-1utr, myo-2p::mCherry]*
XZ1835	*nca-1(ox352)* IV*; sek-1(km4)* X*; yakEx72[unc-17p::sek-1::tbb-2utr-operon-GFP::H2B::cye-1utr, myo-2p::mCherry]*
XZ1861	*nca-1(ox352)* IV*; sek-1(km4)* X*; yakEx73[unc-47p::sek-1::tbb-2utr-operon-GFP::H2B::cye-1utr, myo-2p::mCherry]*
XZ1862	*nca-1(ox352)* IV *; sek-1(km4)* X *; yakEx82[unc-17Hp::sek-1::tbb-2utr-operon-GFP::H2B::cye-1utr, myo-2p::mCherry]*
XZ1863	*egl-30(tg26)* I*; sek-1(km4)* X*; yakEx73[unc-47p::sek-1::tbb-2utr-operon-GFP::H2B::cye-1utr, myo-2p::mCherry]*
XZ1872	*jnk-1(gk7)* I*; sek-1(km4)* X
XZ1873	*pmk-2(qd279 qd171) pmk-1(km25)* IV*; jkk-1(km2)* X
XZ1879	*egl-30(tg26)* I *; sek-1(km4)* X *; yakEx82[unc-17Hp:: sek-1::tbb-2utr-operon-GFP::H2B::cye-1utr, myo-2p::mCherry]*
XZ1902	*egl-30(tg26)* I *; unc-82(e1220)* IV
XZ1937	*sek-1(km4)* X*; yakEx121[hsp-16.2p::sek-1::tbb-2-3' UTR::gld-1 operon*

	linker::gfp::h2b, myo-2p::mCherry]
XZ1938	*egl-30(tg26)* I *; agIs219[T24B8.5p::GFP::unc-54-3'UTR + ttx-3p::GFP::unc-54-3'UTR]* III *atf-7(qd22 qd130)* III
XZ1939	*goa-1(sa734)* I*; sek-1(km4)* X
XZ1942	*tir-1(ky648gf)* III
XZ2054	*eat-16(tm775)* I *; sek-1(km4)* X
XZ2062	*egl-30(js126gf)* I *; sek-1(km4)* X
ZD202	*sek-1(km4)* X*; qdEx8[unc-119p::sek-1::GFP::unc-54-3' UTR + myo-2p::mStrawberry::unc-54-3'UTR]*
ZD318	*agIs29 atf-7(qd22 qd130)* III
ZD442	*agIs29 atf-7(qd22)* III
ZD934	*pmk-2(qd279 qd171) pmk-1(km25)* IV
ZD1020	*pmk-2(qd287)* IV

Table S2. Plasmids and Primers

Gateway entry clones

Plasmid	Details
pADA180	*unc-17Hp* [4-1]
pJH21	*sek-1* cDNA [1-2]
pCFJ150	pDEST5605[4-3]
pCFJ326	*tbb-2utr-operon-GFP::H2B::cye-1utr* [2-3]
pMA23	*unc-17βp* [4-1]
pMH522	*unc-47p* [4-1]
pGH1	*unc-17p* [4-1]
pCM1.56	*hsp-16.2p* [4-1]

Gateway Expression Constructs

Plasmid	Details	Used to make
pJH23	*unc-17p::sek-1::tbb-2utr-operon-GFP::H2B::cye-1utr*	*yakEx72*
pJH24	*unc-47p::sek-1::tbb-2utr-operon-GFP::H2B::cye-1utr*	*yakEx73*
pJH28	*unc-17Hp::sek-1::tbb-2utr-operon-GFP::H2B::cye-1utr*	*yakEx82*
pJH29	*unc-17βp::sek-1::tbb-2utr-operon-GFP::H2B::cye-1utr*	*yakEx83*
pJH46	*hsp-16.2p::sek-1::tbb-2utr-operon-GFP::H2B::cye-1utr*	*yakEx121*

Primers

oJH114	GGGGACAAGTTTGTACAAAAAAGCAGGCTcaATGGAGCGAAAAGGACGTG	F to clone *sek-1* cDNA into [1-2]
oJH115	GGGGACCACTTTGTACAAGAAAGCTGGGTgTCATCGTCGCCAAACAGTG	R to clone *sek-1* cDNA into [1-2]

Table S3. Statistical Tests

Figure	Test	p value
1C	One-way ANOVA and Bonferroni's Multiple Comparison Test<br>WT vs *sek-1(km4)* (ns)	< 0.001

	WT vs *egl-30(tg26)* (p<0.001)<br>*egl-30(tg26)* vs *egl-30(tg26); sek-1(km4)* (p<0.001)<br>*egl-30(tg26)* vs *egl-30(tg26); sek-1(yak42)* (p<0.001)<br>*egl-30(tg26)* vs *egl-30(tg26); unc-82(e1220)* (ns)	
1E	One-way ANOVA and Bonferroni's Multiple Comparison Test<br>WT vs *egl-30(tg26)* (p<0.001)<br>*egl-30(tg26)* vs *egl-30(tg26); sek-1(yak42)* (p<0.001)<br>*egl-30(tg26)* vs *egl-30(tg26); sek-1(km4)* (p<0.001)	< 0.001
1F	One-way ANOVA and Dunnett's Multiple Comparison Test<br>WT vs *sek-1(yak42)* (p<0.001)<br>WT vs *sek-1(km4)* (p<0.001)	< 0.001
1G	One-way ANOVA and Bonferroni's Multiple Comparison Test<br>WT vs *egl-30(js126)* (p<0.001)<br>WT vs *sek-1(km4)* (p<0.001)<br>*egl-30(js126)* vs *egl-30(js126); sek-1(km4)* (p<0.001)	< 0.001
1H	One-way ANOVA and Bonferroni's Multiple Comparison Test<br>WT vs *egl-30(js126)* (p<0.001)<br>*egl-30(js126)* vs *egl-30(js126); sek-1(km4)* (p<0.001)	<0.001
2A	One-way ANOVA and Bonferroni's Multiple Comparison Test<br>*sek-1(km4)* vs *sek-1(km4); qdEx8[unc-119::sek-1(+)]* (p<0.001)	< 0.001
2B	One-way ANOVA and Bonferroni's Multiple Comparison Test<br>*sek-1(km4)* vs *sek-1(km4); yakEx72[unc-17p::sek-1(+)]* (p<0.001)<br>*sek-1(km4)* vs *sek-1(km4); yakEx73[unc-47p::sek-1(+)]* (ns)	<0.001
2D	One-way ANOVA and Bonferroni's Multiple Comparison Test<br>WT vs *egl-30(tg26)* (p<0.001)	<0.001

	egl-30(tg26); sek-1(km4) vs<br>*egl-30(tg26); sek-1(km4); qdEx8[unc-119p::sek-1(+)]*<br>(p<0.001)<br>*egl-30(tg26); sek-1(km4)* vs<br>*egl-30(tg26); sek-1(km4); yakEx72[unc-17p::sek-1(+)]*<br>(p<0.001)<br>*egl-30(tg26); sek-1(km4)* vs<br>*egl-30(tg26); sek-1(km4); yakEx73[unc-47p::sek-1(+)]* (ns)	
2E	Kruskal-Wallis Test and Dunn’s Multiple Comparison Test<br>*egl-30(tg26); sek-1(km4)* vs<br>*egl-30(tg26); sek-1(km4); qdEx8[unc-119p::sek-1(+)]* (p<0.001)<br>*egl-30(tg26); sek-1(km4)* vs<br>*egl-30(tg26); sek-1(km4); yakEx72[unc-17p::sek-1(+)]* (p<0.01)<br>*egl-30(tg26); sek-1(km4)* vs<br>*egl-30(tg26); sek-1(km4); yakEx73[unc-47p::sek-1(+)]* (ns)	< 0.001
2F	One-way ANOVA and Bonferroni's Multiple Comparison Test<br>*sek-1(km4)* vs *sek-1(km4); yakEx121[hsp-16.2p::sek-1(+)]*<br>(p<0.001)	< 0.001
3A	Unpaired t test, two-tailed	< 0.001
3B	One-way ANOVA and Bonferroni's Multiple Comparison Test<br>*egl-30(tg26)* vs *egl-30(tg26); tir-1(tm3036)* (p<0.001)	< 0.001
3C	Unpaired t test, two-tailed	< 0.001
3D	One-way ANOVA and Bonferroni's Multiple Comparison Test<br>*egl-30(tg26)* vs *egl-30(tg26); nsy-1(ok593)* (p<0.001)	< 0.001
3E	One-way ANOVA and Dunnett's Multiple Comparison Test	< 0.001

	WT vs *pmk-1(km25)* (ns)<br>WT vs *pmk-2(qd287)* (p<0.05)<br>WT vs *pmk-2(qd279 qd171) pmk-1 (km25)* (p<0.001)<br>WT vs *pmk-3(ok169)* (p<0.001)	
3F	One-way ANOVA and Dunnett's Multiple Comparison Test<br>*egl-30(tg26)* vs *egl-30(tg26); pmk-1(km25)* (p<0.001)<br>*egl-30(tg26)* vs *egl-30(tg26); pmk-2(qd287)* (p<0.001)<br>*egl-30(tg26)* vs *egl-30(tg26); pmk-2(qd279 qd171) pmk-1 (km25)* (p<0.001)<br>*egl-30(tg26)* vs *egl-30(tg26); pmk-3(ok169)* (p<0.001)	< 0.001
3H	One-way ANOVA and Bonferroni's Multiple Comparison Test<br>*egl-30(tg26)* vs *egl-30(tg26); tir-1(tm3036)* (p<0.001)<br>*egl-30(tg26)* vs *egl-30(tg26); nsy-1(ok593)* (p<0.001)<br>*egl-30(tg26)* vs *egl-30(tg26); sek-1(km4)* (p<0.001)<br>*egl-30(tg26)* vs *egl-30(tg26); pmk-2(qd279 qd171) pmk-1(km25)* (p<0.001)	< 0.001
4A	One-way ANOVA and Bonferroni's Multiple Comparison Test<br>*egl-8(sa47)* vs *egl-8(sa47); sek-1(yak42)* (p<0.001)	< 0.001
4B	One-way ANOVA and Bonferroni's Multiple Comparison Test<br>*sek-1(yak42)* vs *rund-1(tm3622); sek-1(yak42)* (p<0.001)	< 0.001
4C	One-way ANOVA and Bonferroni's Multiple Comparison Test<br>*unc-73(ox317)* vs *unc-73(ox317); sek-1(yak42)* (ns)	< 0.001
4E	One-way ANOVA and Bonferroni's Multiple Comparison Test<br>WT vs *nzIs29[unc-17p::rho-1(G14V)]* (p<0.001)<br>WT vs *nzIs29[unc-17p::rho-1(G14V)]; sek-1(km4)* (p<0.001)	< 0.001

	nzIs29[unc-17p::rho-1(G14V)] vs<br>*nzIs29[unc-17p::rho-1(G14V)]; sek-1(km4)* (ns)	
4F	One-way ANOVA and Bonferroni's Multiple Comparison Test<br>*nzIs29[unc-17p::rho-1(G14V)]* vs<br>*nzIs29[unc-17p::rho-1(G14V)]; sek-1(km4)* (p<0.001)	< 0.001
5B	One-way ANOVA and Bonferroni's Multiple Comparison Test<br>WT vs *sek-1(km4)* (ns)<br>WT vs *nca-1(ox352)* (p<0.001)<br>*nca-1(ox352)* vs *nca-1(ox352); sek-1(km4)* (p<0.01)<br>*nca-1(ox352)* vs *nca-1(ox352); nsy-1(ok593)* (p<0.001)	<0.001
5C	One-way ANOVA and Bonferroni's Multiple Comparison Test<br>WT vs *nca-1(ox352)* (p<0.001)<br>*nca-1(ox352)* vs *nca-1(ox352); nsy-1(ok593)* (p<0.01)<br>*nca-1(ox352)* vs *nca-1(ox352); sek-1(km4)* (p<0.05)	< 0.001
5E	One-way ANOVA and Bonferroni's Multiple Comparison Test<br>*nca-1(ox352)* vs *nca-1(ox352); sek-1(km4)* (p<0.001)<br>*nca-1(ox352); sek-1(km4)* vs<br>*nca-1(ox352); sek-1(km4); qdEx8[unc-119p::sek-1(+)]*<br>(p<0.001)<br>*nca-1(ox352); sek-1(km4)* vs<br>*nca-1(ox352); sek-1(km4); yakEx72[unc-17p::sek-1(+)]*<br>(p<0.01)<br>*nca-1(ox352); sek-1(km4)* vs<br>*nca-1(ox352); sek-1(km4); yakEx73[unc-47p::sek-1(+)]* (ns)<br>*nca-1(ox352); sek-1(km4)* vs	< 0.001

	nca-1(ox352); sek-1(km4); yakEx82[unc-17Hp::sek-1(+)] (ns)<br>*nca-1(ox352); sek-1(km4)* vs<br>*nca-1(ox352); sek-1(km4); yakEx83[unc-17βp::sek-1(+)]*<br>(p<0.001)	
S1A	One-way ANOVA and Bonferroni's Multiple Comparison Test<br>WT vs *egl-30(tg26)* (p<0.01)<br>WT vs *sek-1(km4)* (p<0.001)<br>*egl-30(tg26)* vs *egl-30(tg26); sek-1(km4)* (p<0.001)<br>*sek-1(km4)* vs *egl-30(tg26); sek-1(km4)* (p<0.01)	< 0.001
S1B	One-way ANOVA and Newman-Keuls Multiple Comparison Test<br>WT vs *sek-1(yak42)* (p<0.001)<br>WT vs *sek-1(ag1)* (p<0.001)<br>*sek-1(yak42)* vs *sek-1(ag1)* (ns)	< 0.001
S1C	One-way ANOVA and Bonferroni's Multiple Comparison Test<br>WT vs *egl-30(tg26)* (ns)<br>*egl-30(tg26)* vs *egl-30(tg26); unc-82(e1220)* (p<0.001)	< 0.001
S1D	One-way ANOVA and Bonferroni's Multiple Comparison Test<br>WT vs *sek-1(km4)* (p<0.001)<br>*goa-1(sa734)* vs *sek-1(km4)* (p<0.001)<br>*goa-1(sa734)* vs *goa-1(sa734); sek-1(km4)* (p<0.001)	< 0.001
S1E	One-way ANOVA and Bonferroni's Multiple Comparison Test<br>WT vs *sek-1(km4)* (ns)<br>WT vs *goa-1(sa734)* (p<0.001)<br>*goa-1(sa734)* vs *goa-1(sa734); sek-1(km4)* (p<0.001)	< 0.001
S1F	One-way ANOVA and Bonferroni's Multiple Comparison Test	< 0.001

	WT vs *sek-1(km4)* (p<0.001)<br>WT vs *eat-16(tm775)* (p<0.001)<br>*eat-16(tm775)* vs *eat-16(tm775); sek-1(km4)* (p<0.001)<br>*sek-1(km4) vs eat-16(tm775); sek-1(km4)* (ns)	
S1G	One-way ANOVA and Bonferroni's Multiple Comparison Test<br>WT vs *eat-16(tm775)* (p<0.001)<br>*eat-16(tm775)* vs *eat-16(tm775); sek-1(km4)* (ns)	< 0.001
S2A	One-way ANOVA and Bonferroni's Multiple Comparison Test<br>WT vs *sek-1(km4)* (p<0.001)<br>*sek-1(km4)* vs *sek-1(km4); yakEx82[unc-17Hp::sek-1(+)]* (p<0.05)<br>*sek-1(km4)* vs *sek-1(km4); yakEx83[unc-17βp::sek-1(+)]* (p<0.01)	< 0.001
S2B	One-way ANOVA and Bonferroni's Multiple Comparison Test<br>*egl-30(tg26); sek-1(km4)* vs *egl-30(tg26); sek-1(km4); yakEx82[unc-17Hp::sek-1(+)]* (p<0.001)<br>*egl-30(tg26); sek-1(km4)* vs *egl-30(tg26); sek-1(km4); yakEx83[unc-17βp::sek-1(+)]* (p<0.001)	< 0.001
S2C	One-way ANOVA and Bonferroni's Multiple Comparison Test<br>WT vs *egl-30(tg26)* (p<0.001)<br>*egl-30(tg26); sek-1(km4)* vs<br>*egl-30(tg26); sek-1(km4); qdEx8[unc-119p::sek-1(+)]*<br>(p<0.001)<br>*egl-30(tg26); sek-1(km4)* vs<br>*egl-30(tg26); sek-1(km4); yakEx72[unc-17p::sek-1(+)]*<br>(p<0.001)<br>*egl-30(tg26); sek-1(km4)* vs	< 0.001

	egl-30(tg26); sek-1(km4); yakEx73[unc-47p::sek-1(+)] (ns)<br>*egl-30(tg26); sek-1(km4)* vs *egl-30(tg26); sek-1(km4); yakEx82[unc-17Hp::sek-1(+)]* (p<0.001)<br>*egl-30(tg26); sek-1(km4)* vs *egl-30(tg26); sek-1(km4); yakEx83[unc-17βp::sek-1(+)]* (ns)	
S3A	One-way ANOVA and Bonferroni's Multiple Comparison Test<br>*jnk-1(gk7)* vs *jnk-1(gk7); sek-1(km4)* (p<0.01)<br>*jkk-1(km2) vs pmk-2(qd279 qd171) pmk-1 (km25); jkk-1(km2)* (p<0.05)	< 0.05
S3B	One-way ANOVA and Bonferroni's Multiple Comparison Test<br>WT vs *egl-30(tg26)* (P<0.001)<br>*egl-30(tg26)* vs *egl-30(tg26)*; *jkk-1* (ns)<br>*egl-30(tg26)* vs *egl-30(tg26)*; *jnk-1* (ns)<br>*egl-30(tg26)* vs *egl-30(tg26)*; *atf-7* (ns)	
S3C	One-way ANOVA and Bonferroni's Multiple Comparison Test<br>WT vs *atf-7(qd22)* (p<0.001)<br>WT vs *atf-7(qd22 qd130)* (p<0.001)	< 0.001
S3D	One-way ANOVA	p=0.806
S3F	Unpaired t test, two-tailed	< 0.001
S4A	One-way ANOVA and Bonferroni's Multiple Comparison Test<br>WT vs *nca-1(ox352)* (p<0.001)<br>*nca-1(ox352)* vs *nca-1(ox352); sek-1(km4)* (p<0.001)<br>*nca-1(ox352)* vs *nca-1(ox352); nsy-1(ok593)* (p<0.001)	<0.001
S4B	Kruskal-Wallis Test and Dunn’s Multiple Comparison Test	0.001

	nca-1(ox352);sek-1(km4) vs *nca-1(ox352);sek-1(km4); qdEx8[unc-119p::sek-1(+)]* (ns)<br>*nca-1(ox352);sek-1(km4)* vs<br>*nca-1(ox352);sek-1(km4); yakEx72[unc-17p::sek-1(+)]* (ns)<br>*nca-1(ox352);sek-1(km4)* vs<br>*nca-1(ox352);sek-1(km4); yakEx73[unc-47p::sek-1(+)]* (ns)	
S4C	One-way ANOVA and Bonferroni's Multiple Comparison Test<br>*nca-1(ox352)* vs *nca-1(ox352); sek-1(km4)* (p<0.001)<br>*nca-1(ox352); sek-1(km4)* vs<br>*nca-1(ox352); sek-1(km4); qdEx8[unc-119p::sek-1(+)]*<br>(p<0.001)<br>*nca-1(ox352); sek-1(km4)* vs<br>*nca-1(ox352); sek-1(km4); yakEx72[unc-17p::sek-1(+)]*<br>(p<0.001)<br>*nca-1(ox352); sek-1(km4)* vs<br>*nca-1(ox352); sek-1(km4); yakEx73[unc-47p::sek-1(+)]* (ns)<br>*nca-1(ox352); sek-1(km4)* vs<br>*nca-1(ox352); sek-1(km4); yakEx82[unc-17Hp::sek-1(+)]*<br>(p<0.001)<br>*nca-1(ox352); sek-1(km4)* vs<br>*nca-1(ox352); sek-1(km4); yakEx83[unc-17βp::sek-1(+)]*<br>(p<0.001)	<0.001

Figure S1

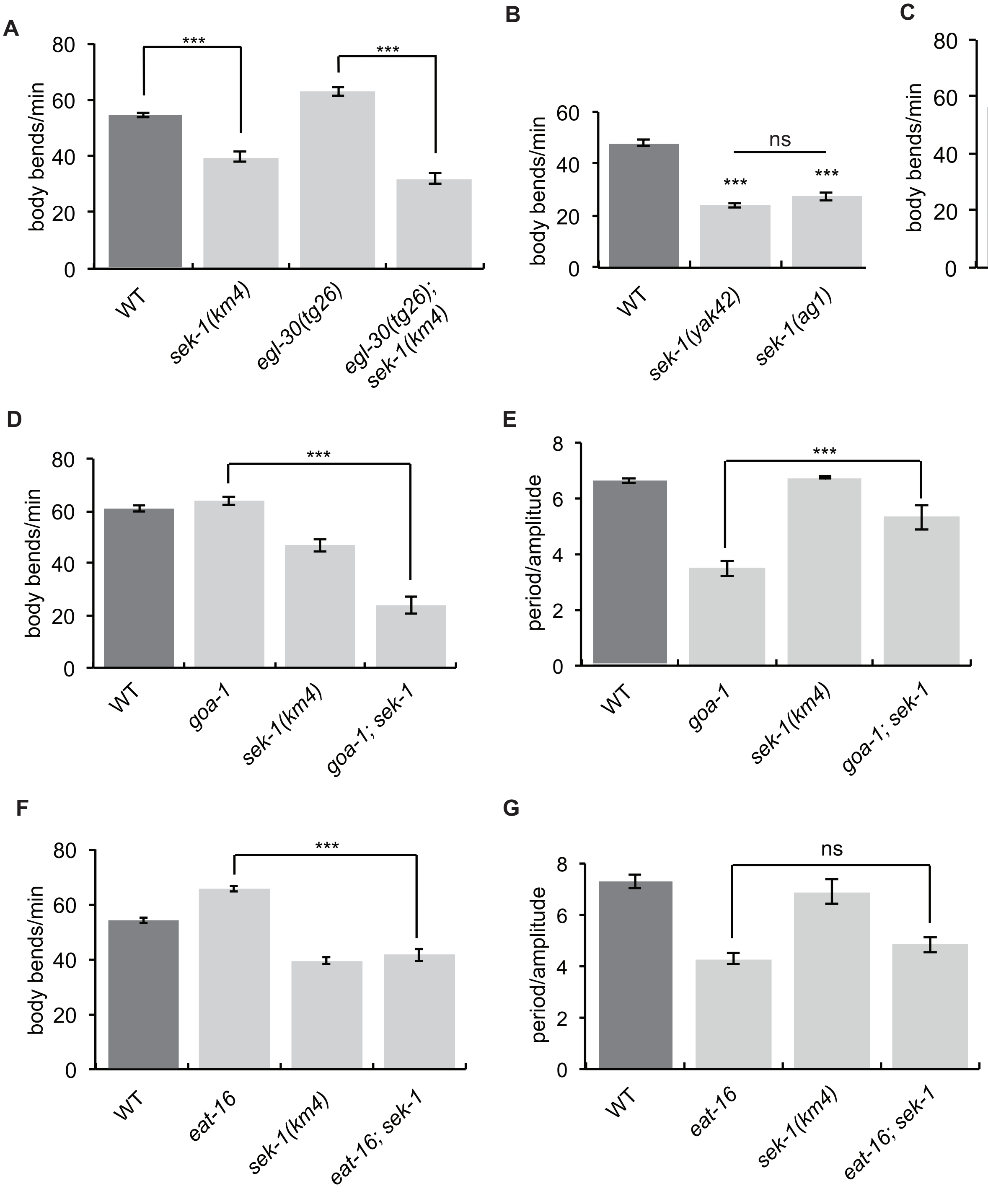

Figure S1. *sek-1* interacts with Gq and Go mutants

(A) The *sek-1(km4)* mutation suppresses the hyperactive locomotion of the activated Gq mutant *egl-30(tg26)*. ***, p<0.001, error bars = SEM, n=20.

(B) *sek-1(ag1)* mutant animals have slow locomotion. ***, p<0.001, error bars = SEM, n=10.

(C) The *unc-82(e1220)* mutation reduces the locomotion rate of the activated Gq mutant *egl-30(tg26).* ***, p<0.001, error bars = SEM, n= 20.

(D) *sek-1(km4)* suppresses the hyperactivity of *goa-1(sa734).* ***, p<0.001, error bars = SEM, n=20.

(E) *sek-1(km4)* suppresses the loopy waveform of *goa-1(sa734)*. ***, p<0.001, error bars = SEM, n=5.

(F) *sek-1(km4)* suppresses the hyperactivity of *eat-16(tm775).* ***, p<0.001, error bars = SEM, n=20.

(G) *sek-1(km4)* does not suppress the loopy waveform of *eat-16(tm775).* ns, p>0.05, error bars = SEM, n=5.

Figure S2

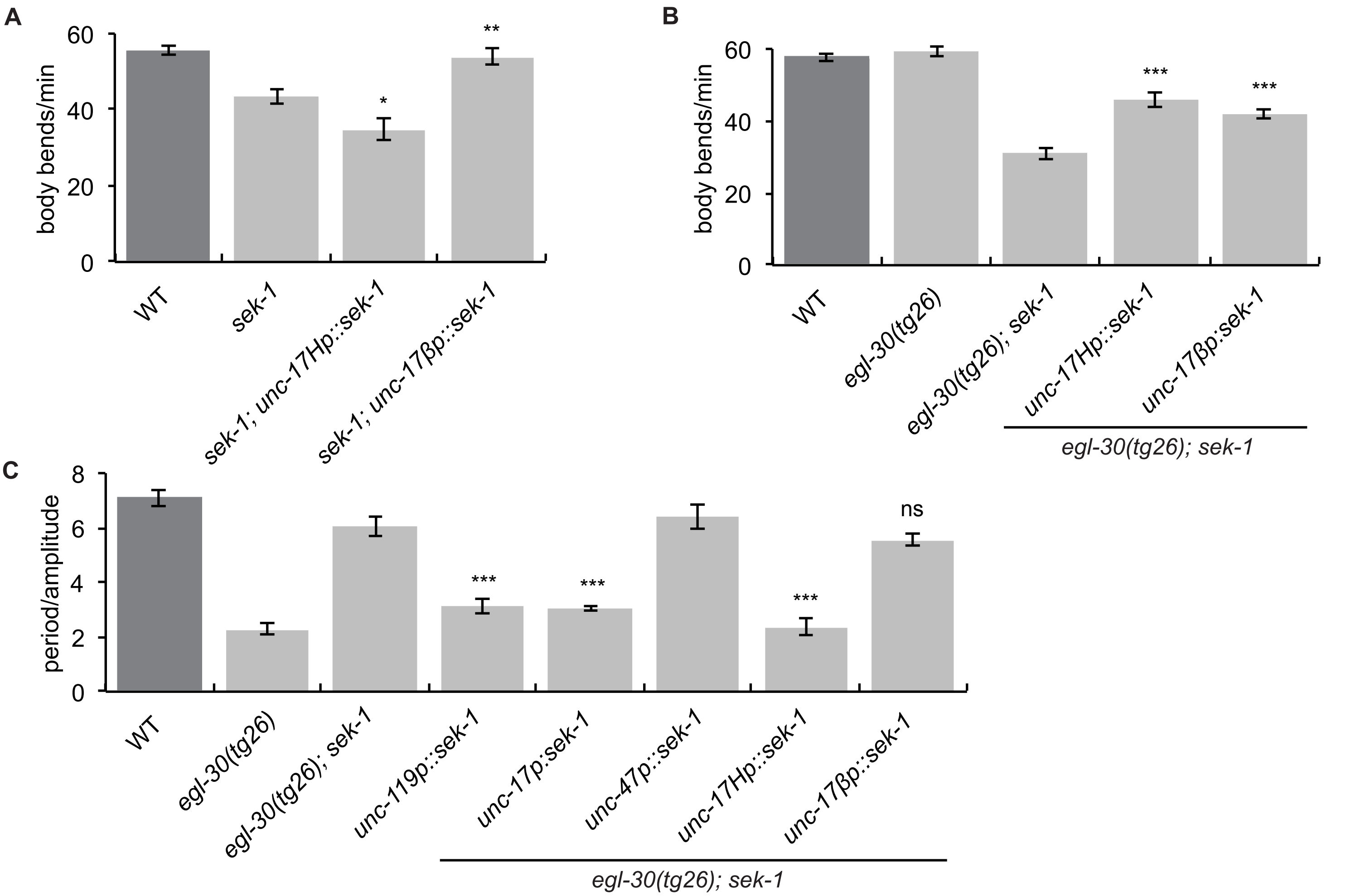

Figure S2. *sek-1* acts in both head acetylcholine neurons and acetylcholine motorneurons

(A) *sek-1* acts in acetylcholine motorneurons to modulate locomotion rate. The *sek-1* WT cDNA driven by the *unc-17β* acetylcholine motorneuron promoter [*unc-17βp::sek-1(+)*] rescues the slow locomotion phenotype of *sek-1(km4)* worms, but *sek-1* expression in head acetylcholine neurons using the *unc-17H* promoter [*unc-17Hp::sek-1*(+)] does not rescue. **, p< 0.01; *, p<0.05 compared to *sek-1*. Error bars = SEM, n=20.

(B) *sek-1* acts in both head acetylcholine neurons and acetylcholine motorneurons to modulate the locomotion rate of the activated Gq mutant *egl-30(tg26). egl-30(tg26) sek-1(km4)* worms expressing either *unc-17Hp::sek-1(+)* or *unc-17βp::sek-1(+)* have an increased locomotion rate compared to *egl-30(tg26) sek-1.* ***, p<0.001 compared to *egl-30(tg26) sek-1.* Error bars = SEM, n=20.

(C) *sek-1* acts in head acetylcholine neurons to modulate the loopy waveform of the activated Gq mutant *egl-30(tg26)*. *egl-30(tg26) sek-1(km4)* worms expressing *unc-17Hp::sek-1(+)* are loopy like *egl-30(tg26)*, but *egl-30(tg26) sek-1(km4*) worms expressing *unc-17βp::sek-1(+)* are similar to *egl-30(tg26) sek-1.* ***, p<0.001; ns, p>0.05 compared to *egl-30(tg26) sek-1*. Error bars = SEM, n=5.

Figure S3

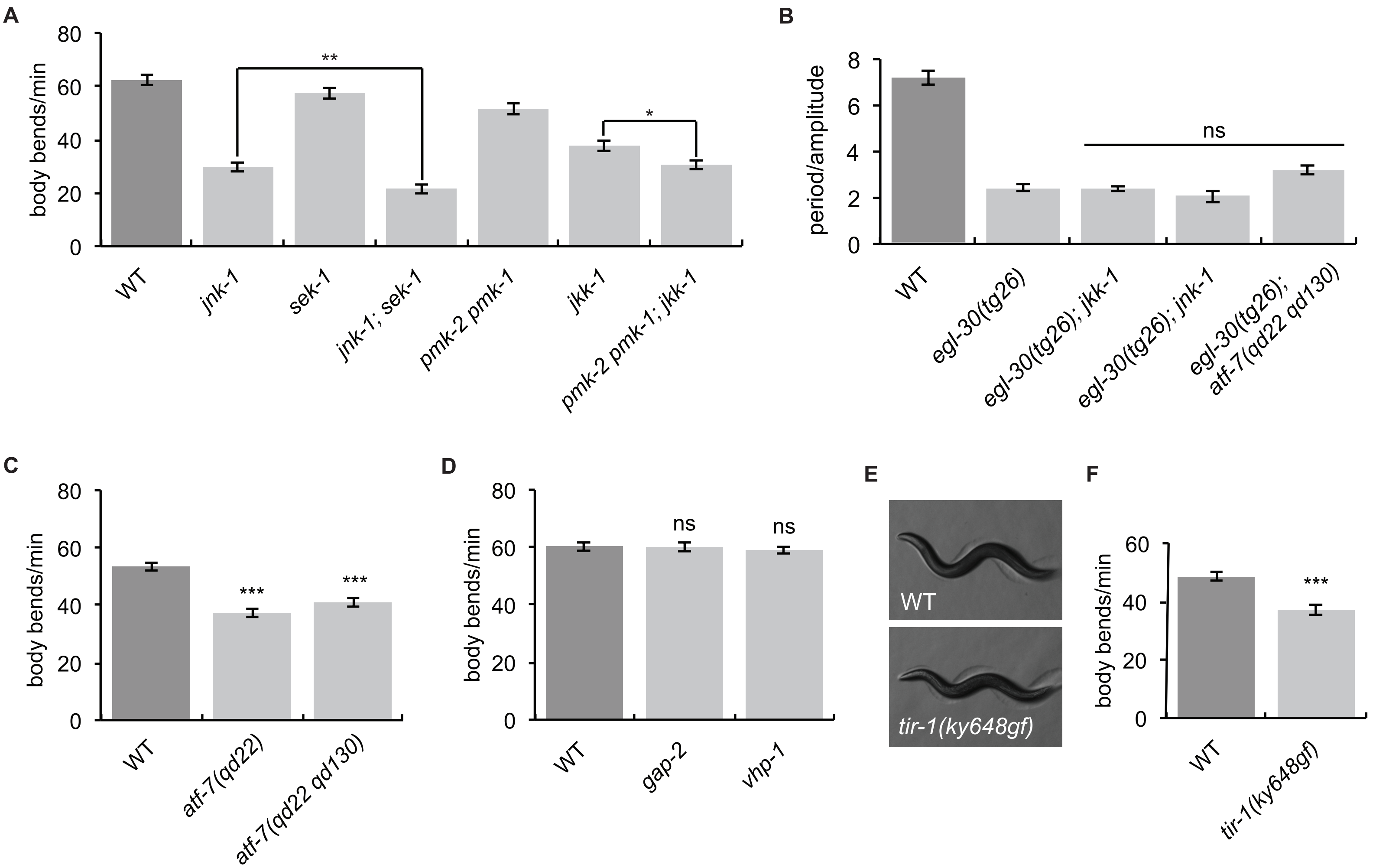

Figure S3. Locomotion of p38 and JNK MAPK pathway mutants

(A) *jkk-1* and *jnk-1* act in parallel to *sek-1* and *pmk-2 pmk-1*. The *jnk-1(gk7) sek-1(km4)* double mutant and *pmk-2(qd279 qd171) pmk-1(km25) jkk-1(km2)* triple mutants move more slowly than the respective individual mutants. **, p< 0.01, *, p<0.05. Error bars = SEM, n=20.

(B) Mutations in *jkk-1, jnk-1,* and *atf-7* do not suppress the loopy waveform of the activated Gq mutant *egl-30(tg26).* ns, p>0.05, error bars = SEM, n=5.

(C) Worms with gain-of-function or loss-of-function alleles of *atf-7* are slower than wild-type worms. ***, p< 0.001, error bars = SEM, n=20.

(D) Worms lacking *gap-2* and *vhp-1* move like wild-type worms. Neither *gap-2(tm478)* nor *vhp-1(sa366)* confers a slow locomotion phenotype. ns, p>0.05 compared to WT. Error bars = SEM, n=20.

(E-F) *tir-1(ky648gf)* animals do not have loopy or hyperactive locomotion. *tir-1(ky648gf)* worms have wild-type posture and are slower than wild-type animals. ***, p< 0.001, error bars = SEM, n=20.

Figure S4

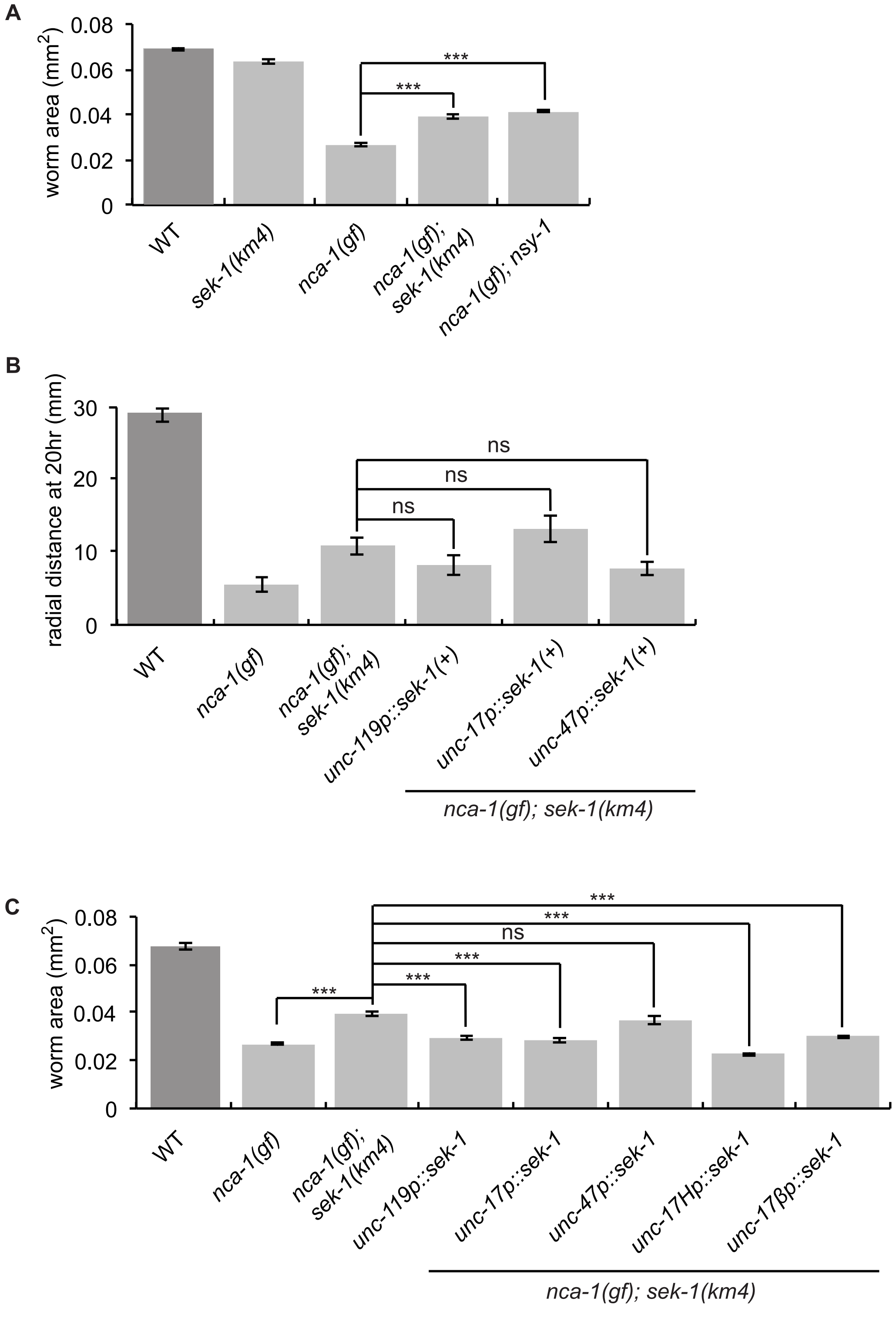

Figure S4. *sek-1* and *nsy-1* weakly suppress *nca-1(gf)*

(A) Mutations in *sek-1* and *nsy-1* suppress the small body size of *nca-1(ox352)* mutant worms. ***, p<0.001, error bars = SEM, n=10.

(B) None of the neuronal *sek-1* rescuing constructs reverse the radial locomotion phenotype of *nca-1(gf) sek-1(km4)* animals. ns, p>0.05. Error bars = SEM, n=19-24.

(C) *sek-1* acts in both head acetylcholine neurons and acetylcholine motorneurons to control the body size of *nca-1(gf)*. *nca-1(ox352) sek-1(km4)* worms expressing *sek-1* in all neurons (*unc-119p::sek-1(+)*), acetylcholine neurons (*unc-17p::sek-1(+)*), head acetylcholine neurons (*unc-17Hp::sek-1(+)*), or acetylcholine motorneurons (*unc-17βp::sek-1(+)*) have a similar size to *nca-1(gf),* but *nca-1(ox352) sek-1(km4)* worms expressing sek-1 in GABA neurons (*unc-47p::sek-1(+)*) are similar to *nca-1(gf) sek-1.*
***, p< 0.001; ns, p>0.05. Error bars = SEM, n=7-10.
